# Supplementary material for: Exploring the impacts of the 2012 Health and Social Care Act reforms to commissioning on clinical activity in the English NHS: a mixed methods study of cervical screening
Source: BMJ Open. 2019 Apr 14;9(4):e024156. doi: 10.1136/bmjopen-2018-024156 (PMC6500278; doi:10.1136/bmjopen-2018-024156)
Supplement: Supplementary file 1 [file bmjopen-2018-024156supp001.pdf]

Technical Appendix

Algebraic expressions for the estimated models

Triple-difference model

The triple-difference model can be written algebraically as:

$$\boldsymbol{y_{cjt} = \alpha + \phi L_j + \theta \pi_c + \rho L_j \pi_c + \sum_{t=2}^{T} \delta_t + \sum_{t=2}^{T} \psi_t \pi_c + \sum_{t=2}^{T} \gamma_t L_j + \beta L_j \pi_c D_t^* + \varepsilon_{cjt}}$$

in which y_{cjt} is the value of indicator c in practice j and year t, L_j is a binary indicator for practices located in exposed areas, π_c is a dummy variable for the intervention indicator, $t_{2 \dots} t_T$ are dummy variables for years, and $\boldsymbol{D_t^*}$ is a dummy variable taking a value of one in post-implementation period. The coefficient of interest is β, the coefficient on the three-way interaction between the exposed area, the intervention indicator and the post-implementation period.

Lagged Dependent Variable model

The Lagged Dependent Variable model can be written algebraically as:

$$\boldsymbol{y_{cjt} = \alpha + \phi L_j + x \pi_c + \rho L_j \pi_c + \sum_{t=t^*}^{T} \delta_t + \sum_{t=t^*}^{T} \psi_t \pi_c + \sum_{k=1}^{t^*-1} \mu_k y_{cjk} + \sum_{k=1}^{t^*-1} \vartheta_k \pi_c y_{cjk} + \varepsilon_{cjt}}$$

in which y_{cjt} is the value of indicator c in practice j and year t, L_j is a binary indicator for practices located in exposed areas, π_c is a binary indicator for the intervention indicator, and y_{cjk} are lagged values of the dependent variable in the pre-period ending at $t=t^*-1$. The model is estimated on data from the post period only and the ρ coefficient on the $L_j \pi_c$ term is the coefficient of interest.
