# Supplementary material for: Exploring the impacts of the 2012 Health and Social Care Act reforms to commissioning on clinical activity in the English NHS: a mixed methods study of cervical screening
Source: BMJ Open. 2019 Apr 14;9(4):e024156. doi: 10.1136/bmjopen-2018-024156 (PMC6500278; doi:10.1136/bmjopen-2018-024156)
Supplement: Author's manuscript [file bmjopen-2018-024156.draft_revisions.pdf]

BMJ Open is committed to open peer review. As part of this commitment we make the peer review history of every article we publish publicly available.

When an article is published we post the peer reviewers' comments and the authors' responses online. We also post the versions of the paper that were used during peer review. These are the versions that the peer review comments apply to.

The versions of the paper that follow are the versions that were submitted during the peer review process. They are not the versions of record or the final published versions. They should not be cited or distributed as the published version of this manuscript.

BMJ Open is an open access journal and the full, final, typeset and author-corrected version of record of the manuscript is available on our site with no access controls, subscription charges or pay-per-view fees (http://bmjopen.bmj.com).

If you have any questions on BMJ Open's open peer review process please email info.bmjopen@bmj.com

BMJ Open

Exploring the impacts of the 2012 Health and Social Care Act reforms to commissioning upon clinical activity in the English NHS: A mixed methods study of cervical screening

Journal:	*BMJ Open*
Manuscript ID	bmjopen-2018-024156
Article Type:	Research
Date Submitted by the Author:	11-May-2018

Complete List of Authors:	Hammond, Jonathan; University of Manchester, Division of Population Health, Health Services Research, and Primary Care; School of Health Science<br>Mason, Thomas<br>Sutton, Matt; University of Manchester<br>Hall, Alex; University of Manchester, Division of Nursing, Midwifery & Social Work; School of Health Sciences<br>Mays, Nicholas; London School of Hygiene and Tropical Medicine, Public Health and Policy<br>Coleman, Anna; University of Manchester, Centre for Primary Care<br>Allen, Pauline; Health Services Research Unit<br>Warwick-Giles, Lynsey; University of Manchester Institute of Population Health<br>Checkland, Kath; University of Manchester, Institute of Population Health; Centre for Biostatistics

Keywords:	NHS, commissioning, mixed methods, health policy, screening

SCHOLARONE™
Manuscripts

Exploring the impacts of the 2012 Health and Social Care Act reforms to commissioning upon clinical activity in the English NHS: A mixed methods study of cervical screening

Authors

- *Dr Jonathan Hammond [Corresponding author]*

Division of Population Health, Health Services Research, and Primary Care; School of Health Science; Faculty of Biology, Medicine and Health; Williamson Building (6th floor), The University of Manchester, Manchester, M13 9PL, UK

Email: jonathan.hammond@manchester.ac.uk; Tel.: 0161 2757648

- *Dr Thomas Mason*

Centre for Health Economics; Division of Population Health, Health Services Research, and Primary Care; School of Health Science; Faculty of Biology, Medicine and Health; The University of Manchester, Manchester, UK

- *Prof Matt Sutton*

Centre for Health Economics; Division of Population Health, Health Services Research, and Primary Care; School of Health Science; Faculty of Biology, Medicine and Health; The University of Manchester, Manchester, UK

- *Dr Alex Hall*

Manchester Institute for Collaborative Research on Ageing; School of Social Sciences; Faculty of Humanities; The University of Manchester, Manchester, UK

- *Prof Nicholas Mays*

Department of Health Services Research and Policy; Faculty of Public Health and Policy; London School of Hygiene and Tropical Medicine, London, UK

- *Dr Anna Coleman*

Division of Population Health, Health Services Research, and Primary Care; School of Health Science; Faculty of Biology, Medicine and Health; The University of Manchester, Manchester, UK

- *Prof Pauline Allen*

Department of Health Services Research and Policy; Faculty of Public Health and Policy; London School of Hygiene and Tropical Medicine, London, UK

- *Dr Lynsey Warwick-Giles*

Division of Population Health, Health Services Research, and Primary Care; School of Health Science; Faculty of Biology, Medicine and Health; The University of Manchester, Manchester, UK

- *Prof Kath Checkland*

Division of Population Health, Health Services Research, and Primary Care; School of Health Science; Faculty of Biology, Medicine and Health; The University of Manchester, Manchester, UK

Word count

5054

For peer review only

ABSTRACT

Objectives: Explore the impact on cervical screening activity of the Health and Social Care Act (HSCA) reforms to commissioning responsibilities introduced in the English NHS in April 2013.

Methods: Sequential mixed methods. Qualitative: semi-structured interviews (with commissioners, managers, clinicians, and senior administrative staff from Clinical Commissioning Groups (CCGs), Local Authorities (LAs), service providers) and observations of commissioning meetings in two metropolitan areas of England. Quantitative: triple difference-in-differences analysis of national administrative data. Variability in the expected effects of HSCA on commissioning was measured by comparing CCGs working with one LA compared with CCGs working with multiple LAs. To control for unmeasured confounders, differential changes over time in cervical screening rates (amongst women aged 25-64 years) between CCGs more and less likely to have been affected by HSCA commissioning disruption were compared with another outcome – unassisted birth rates – largely unaffected by HSCA changes.

Results: Interviewees suggested cervical screening commissioning and provision had become more complex and 'fragmented', with responsibilities less certain, following the HSCA. Interviewees predicted because of this there would be reductions in cervical screening rates, concentrated in some areas. Quantitative findings supported these predictions: cervical screening rates decreased following the HSCA introduction. Areas where CCGs deal with multiple LAs experienced a larger decline in cervical screening rates (1.4 percentage points) compared with those dealing with only one LA (1.0 percentage points). Over the same period, unassisted deliveries decreased by 1.6 percentage points and 2.0 percentage points respectively in the two groups.

Conclusions: Arrangements for commissioning and delivering cervical screening were disrupted by the HSCA. This reduced screening rates particularly in the most affected areas. This study's findings are strengthened by the cumulative effect of the novel approach to sequential mixed methods adopted. Minimise ambiguity over commissioning responsibilities as a consequence of large-scale health system reform is important.

Keywords:

Screening; Health policy; Mixed methods; Commissioning; NHS

For peer review only

ARTICLE SUMMARY

Strengths and limitations of this study:

- Few studies have investigated in detail the impacts of large-scale health system change.
- This study combines detailed qualitative data exploring impacts on the system with quantitative exploration of important outcomes, supporting causal inference.
- We developed a quantitative measure for assessing the extent of disruption to the English NHS' commissioning system as a result of the 2012 Health and Social Care Act.
- We found that cervical screening rates decreased more post-Act in areas that had experienced higher levels of disruption.

INTRODUCTION

Centrally-driven, structural re-organisations of publicly financed health care systems are frequently employed with the objective of improving health care delivery, and thus population health outcomes, whilst reducing or containing costs (Best et al. 2012). Such endeavours can be disruptive and expensive (Edwards 2010). It is important to understand what possible impacts these reorganisations have in order to understand their value (Lale and Temple 2016).

In the English NHS, attempts to evaluate the impact of reorganisations have typically used operational indicators (e.g. bed availability, numbers of staff) and measures of clinical activity because their improvement was the stated goal of government policy (e.g. The NHS Plan, Department of Health 2000). Other studies have attempted to measure the impacts of reforms by measuring their effects on prices, quality and quantity of provision (Propper 1996). Most studies have relied upon quantitative analysis of measures that were explicitly targeted by policy reforms. There is a need for approaches which combine qualitative and quantitative methods to generate a deeper understanding of the impacts of structural reorganisation (Barbour 1999).

The most recent structural reorganisation of the English NHS, the Health and Social Care Act (2012) (hereafter 'HSCA' or 'the Act'), was introduced in April 2013 implementing wide-ranging reforms to commissioning in the health and social care system in England. In this paper, we explore whether these reforms have affected measures of clinical activity in cervical screening services in order to explore whether changes to the commissioning of these services, implemented as part of the Act, affected uptake. This paper goes beyond the typical analyses of large-scale NHS reorganisation outlined above by utilising a novel mixed methods approach. Our 'bottom up' qualitative analysis

For peer review only - http://bmjopen.bmj.com/site/about/guidelines.xhtml

allowed us to identify problematic issues associated with the HSCA for those working on the ground in the health and care system. Both the understanding of disruption to established commissioning arrangements and the identification of cervical screening as a clinical activity potentially susceptible to the consequences of this occurred through this process. We then developed an appropriate quantitative component specifically to test these issues and better understand the impacts of the policy. Together, these analyses allow us to infer causation.

The HSCA and changes to cervical screening commissioning

The HSCA is regarded as one of the most wide-ranging legislative reforms of the English NHS (Ham et al. 2015). Primary Care Trusts (PCTs), 152 organisations previously responsible for the commissioning of primary, community and secondary health services from providers on behalf of local populations, were abolished. Their commissioning functions were split between three groups of organisations: 211 (now 208) newly-created Clinical Commissioning Groups (CCGs), membership organisations constituted by GP (family doctor) practices, given responsibility for commissioning services for their local populations; NHS England (originally NHS Commissioning Board), a new arm's-length governmental body with responsibility for authorising and assessing CCGs, and commissioning some services at a national level; and top-tier and single-tier elected local authorities (LAs), which took on responsibility for the majority of public health services for the first time since 1974. In addition, Public Health England (PHE) was created as an executive agency of the Department of Health, to unify the diverse public health profession and provide expert support for public health services.

In some service areas, the transfer of responsibility was relatively straightforward (e.g. the commissioning of routine orthopaedic surgery was passed from PCTs to CCGs with minimal alteration to the bundle of services involved). In other service areas, the transfers were much more complex, particularly in the commissioning of national screening programmes and sexual health services, as a result of changes to public health commissioning. Pre-HSCA, national screening programmes and sexual health services were both commissioned by PCTs. Cervical screening was provided by GP practices, which received additional funding linked to levels of activity (Peckham et al. 2015). Post-HSCA, responsibility for public health services, including most sexual health services, was transferred to LAs. The underlying programme theory (i.e. explicit expectations about how a policy or course of action will work; Weiss 1998) was that LAs would be better placed to address wider determinants of health and wellbeing than the NHS because by incorporating public health commissioning responsibilities into their portfolio they could develop more joined up services with areas such as transport and housing (Department of Health 2010). NHS England took responsibility for commissioning national screening programmes, under the public health functions agreement

For peer review only

between the Secretary of State for Health and NHS England (Ham et al. 2015). NHS England's regional teams are responsible for commissioning screening programmes, supported by PHE employees 'embedded' within their screening and immunisation teams (NHS England and Public Health England 2013). There is no identifiable underlying programme theory for this specific change to screening programme commissioning. However, it is notable that, in contrast to the emphasis placed upon localism associated with the creation of CCGs and with the transfer of public health to LAs, screening commissioning became more centralised as a consequence of the HSCA.

Table 1 shows the organisations with responsibilities of relevance to the commissioning of sexual health and cervical screening services, pre and post-HSCA. It illustrates how responsibility for such services, previously commissioned by PCTs, are now split across different agencies. This increased complexity and fragmentation of responsibilities has the potential to disrupt service commissioning (Peckham et al. 2017).

Table 1: Organisations of significance to the commissioning of cervical screening pre and post HSCA

Pre-HSCA	
Primary Care Trusts	Responsible for all public health commissioning, including sexual health services; Responsible for commissioning national screening programmes, including cervical screening
UK National Screening Committee	Provision of advice and support to NHS organisations on population screening programmes
Post-HSCA	
Local Authorities	Responsible for most public health commissioning, including most sexual health services (contraception over and above GP contract, testing and treatment of sexually transmitted infections, sexual health advice and promotion)
NHS England (NHSE)	Responsible for commissioning national screening programmes, including cervical screening, and some sexual health services (notably contraception through GP contract, and HIV treatment)
Public Health England (PHE)	Hosts UK National Screening Committee secretariat, which retains the same role; PHE staff embedded in sub-regional NHSE screening and immunisation teams to provide expertise and 'leadership'
Clinical Commissioning Groups (CCGs)	Responsible for improving clinical outcomes for their patients and prevent premature death (as part of the NHS Outcomes Framework, which CCGs are held to account on by NHSE); GP members are funded to carry out cervical screening through the GP Contract

For peer review only

This paper comes from a study designed to foster emergent interplay between qualitative and quantitative work. Our focus on cervical screening was not established at the initial project design stage but driven by findings from broader qualitative research into post-HSCA sexual health commissioning arrangements and screening activity (Guest 2013). This prompted us to consider a quantitative exploration of predictions made by our interviewees relating to cervical screening activity.

METHODS

Study context and design

This study forms part of a longitudinal research project, conducted between January 2015 and December 2017, into the effect of the HSCA upon the commissioning system in England. We combined a qualitative and quantitative exploration of the commissioning of services in two large, socioeconomically diverse metropolitan areas of England with a national level quantitative study of commissioning outcomes. We used a sequential mixed methods approach in which qualitative data collection and analysis was used to shape further quantitative analysis using routinely available data. We therefore present the qualitative and quantitative methods and findings sequentially, and integrate them in the Discussion section.

Qualitative component

Setting, participants, sampling and data collection

The qualitative component took place between March 2015 and August 2017, focussing on two metropolitan ‘health economies’ covering a geographical population and a group of commissioning organisations and providers with close operational links. Across both areas, we conducted 93 interviews (each typically an hour in length), which related to sexual health commissioning, with clinical and non-clinical commisioners, managers, clinicians, and senior administrative staff from CCGs, LAs, service providers and third sector organisations. Organisations and participants were sampled purposively for variation in type and role. We identified participants through organisational websites, personal contacts, and through ‘snowballing’ in which we asked participants to recommend other potential participants. Additionaly, eight hours of meetings of an inter-organisaional sexual health co-ordinating group involving sexual health commissioners and providers were observed in one of the areas.

Interviews focused upon the commissioning system pre- and post-HSCA, exploring continuities and changes to personal and organisational roles, key issues and challenges, accountability and performance management, interorganisational relationships and communication, and

For peer review only - http://bmjopen.bmj.com/site/about/guidelines.xhtml

commissioning decision-making. Interviews took place either in person (usually in participants' offices) or over the telephone. All interviews were audio-recorded and all interviewees were provided with written information about the study before consenting to participate.

Data analysis

Audio recordings of interviews were transribed verbatim, and observational notes from meetings were produced contemporaneously. Transcripts and observational notes were imported into NVivo 10 software and analysed according to the early phases of thematic analysis (Braun and Clarke 2006). Our findings (see below) contained some predictions made by participants regarding cervical screening activity as a consequence of the Act. This prompted us to utilise our mixed methods approach to explore these predictions, and hence we developed complementary quantitative analysis, which we now describe.

Quantitative component

The interviewees told us that the HSCA had introduced confusion over responsibility into the commissioning of cervical screening services. We wished to test whether or not this had an impact on cervical screening rates. As the Act was implemented in all areas simultaneously, we sought to identify a measure of variability in the extent to which the Act would have been expected to affect commissioning in each area. One of the features of the post-HSCA system was that some, but not all CCGs were established which crossed LA boundaries. Some CCGs related to as many as three separate LAs. As each LA developed its own approach to cervical screening provision in local sexual health clinics, we explored the possibility that GP practices located in CCGs which had to work with more than one LA might experience a reduction in screening rates compared with practices located in CCGs which had only to deal with one LA. We compare the demographic characteristics of these two groups in Table 2. The 89 CCGs needing to deal with more than LA had a slightly older population profile than the 119 CCGs who needed to deal with only one LA.

For peer review only

Table 2: CCG demographic characteristics depending on the number of Local Authorities that the CCG needs to work with

	One Local Authority	More than one Local Authority
Number of CCGs	119	89
Population (millions)	29.5	26.9
Female	50.0%	50.5%
Aged 0-9	12.3%	11.4%
Aged 10-19	11.2%	11.2%
Aged 20-39	30.0%	24.8%
Aged 40-59	26.5%	27.7%
Aged 60-79	16.0%	19.7%
Aged 80 and over	4.1%	5.3%

Because cervical screening rates may be influenced by other factors that we cannot observe and change over time in different ways between the two groups of CCGs, we also compared screening rates with an outcome that was likely to have been unaffected by the introduction of HSCA. We used unassisted births (i.e. uncomplicated deliveries which did not require any intervention) as a percentage of all maternal deliveries as the indicator for comparison, as the commissioning of maternity services was largely unchanged by the Act.

We applied a triple difference-in-differences approach. The triple difference represents (the change over time in cervical screening rates for CCGs working with only one LA minus the change over time in cervical screening rates for CCGs working with more than one LA) minus (the change over time in unassisted birth rates for CCGs working with only one LA minus the change over time in unassisted birth rates for CCGs working with more than one LA).

The intervention indicator is the percentage of women aged between 25 and 64 years who had received a cervical screening test in the preceding five years. This indicator was produced using annual, practice-level data from the Quality and Outcomes Framework (QOF) for 2009-10 to 2015-16. The comparison indicator is unassisted births as a percentage of all maternal deliveries. This indicator was produced using operation codes in Hospital Episode Statistics (HES) for 2009-10 to 2015-16. We aggregated the spell-level data by general practice and financial year.

For peer review only - http://bmjopen.bmj.com/site/about/guidelines.xhtml

The key assumption underpinning the triple difference estimator is that, conditional on the other variables in the model, the differences in the changes over time in the intervention indicator between the exposed and the control areas would have been the same as the differences in the changes over time in the comparison indicator between the exposed and control areas in the absence of the intervention. This is a more complex version of the 'parallel trends' assumption required for the double-difference, or difference-in-differences, estimator (Imbens and Wooldridge 2009). A popular test of this assumption in the double-difference case is that there are parallel trends over time in the outcomes in the intervention and comparison group in the pre-intervention period. For our triple-difference case, we applied the equivalent of this test using two alternative approaches. First, we estimated the triple-difference model using data from the pre-intervention period only with a 'placebo' time intervention point equal to one for 2011-12 and 2012-13. Second, we ran a triple-difference model in the pre-period only and tested the joint significance of interactions between the year effects and the binary variable representing the combination of exposed area and treated indicator.

We also used the lagged dependent variable (LDV) estimator. This estimator provides less biased estimates of treatment effects when the assumption of parallel pre-trends does not hold (O'Neill et al. 2016). We set up a triple difference-in-differences LDV model, which includes: dummy variables for year; values of the dependent variable in the pre-intervention period; a dummy variable classifying practices depending on whether they are located in CCGs working with more than one LA; interactions between year and condition dummies; interactions between values of the dependent variable in the pre-intervention period and the condition dummy; an interaction between the dummy variable classifying practices depending on whether they are located in CCGs working with more than one LA and the condition dummy. This can be written algebraically as:

$$\boldsymbol{y_{cjt} = \alpha + \phi L_j + x\pi_c + \rho L_j \pi_c + \delta_1 t_1 + \cdots + \delta_T t_T + \beta_1 y_{cjT-2} + \cdots + \beta_4 y_{cjT-5} + \psi_1 t_1 \pi_c + \cdots + \psi_T t_T \pi_c + a_1 y_{cjT-2} \pi_c + \cdots + a_4 y_{cjT-5} \pi_c + \varepsilon_{cjt}}$$

in which y_{cjt} is the value of indicator c in practice j and year t, L_j is a binary indicator for practices located in exposed CCGs, π_c is a binary indicator for the intervention indicator, $t_{1\ldots}t_T$ are dummy variables for years, and $y_{cjT-2}\ldots y_{cjT-5}$ are lagged values of the dependent variable in the pre-period. This model is estimated only on data in the post-intervention period.

We estimated the regression models in Stata 14.1 using dummy variable least squares regression with fixed effects for practice-indicator combinations. We clustered the standard errors at the GP practice level.

For peer review only - http://bmjopen.bmj.com/site/about/guidelines.xhtml

RESULTS

Qualitative findings

Our analysis identified two main themes – differing perspectives on budgets and responsibilities, and potential impacts on cervical screening rates – both of which relate directly to confusion surrounding the commissioning and provision of cervical screening post-HSCA.

Differing perspectives on budgets and responsibilities

Before the HSCA, both cervical screening and sexual health services were commissioned by PCTs. As one screening and immunisations lead outlined, cervical screening tests (sometimes referred to as smear tests) were provided by GP practices, but patients could usually also have them at sexual health clinics [17685, NHSE, Area 2, Dec 2016]. The HSCA separated the budgets for cervical screening and sexual health, meaning that the LA budget and responsibility for sexual health did not extend to cervical screening. One LA public health consultant reported that, in spite of this, PHE was sending letters to patients explicitly stating that they could choose to attend either their GP practice or their local sexual health clinic for their cervical screening test. This highlights confusion regarding commissioning arrangements and budgetary responsibility:

> Public Health England were writing around to people saying …you're due your smear, you can go to your general practice or you can go to your local sexual health clinic. And we said, but we don't have the money for them to do that, they can't come here routinely unless you're going to pay us for that. Public Health England, the screening people, they have the money to pay for the smears. But in all the moving around of the budgets, the money for smears that were taken outside general practice doesn't seem to be anywhere [8384, LA, Area 1, Nov 2015].

One participant from NHS England offered a different perspective. He argued that the public health budget of each LA reflected the levels of cervical screening activity that had taken place in its sexual health clinics pre-HSCA. However, this is not clear because, in the past, the funding was not "disaggregated" [4058, NHSE, Area 1, Jun 2015]. Therefore, it is not possible to establish what the pre-HSCA sexual health component of the public health budget covered.

> …they [LAs] think they're not being paid for it [cervical screening]. But actually in truth whatever they were doing at the point of transition if they were doing loads of cervical smears they were just doing loads of cervical smears, so they had the money. There wasn't a problem when they were doing them before, it's just the money wasn't

For peer review only - http://bmjopen.bmj.com/site/about/guidelines.xhtml

> disaggregated. However local authorities have been put under significant pressure in their public health teams to reduce their budgets. So these kinds of things are examples where you can say it's not our responsibility so therefore we're taking that element out [4058, NHSE, Area 1, Jun 2015].

The above quote illustrates a phenomenon reported by a number of participants that LAs have re-procured their sexual health services and have taken a position that they will not commission their sexual health provider(s) to do routine cervical screening, because it is not their commissioning responsibility. However, as one member of a screening team in Area 2 illustrated, NHSE is also reluctant to commission sexual health services to provide cervical screening, seemingly because of administrative challenges relating to numerous low-value contracts with providers:

> So cervical screening we could go to every sexual health provider and have a separate contract. The difficulty again becomes around commissioning capacity. So I think we've got [x] local authorities, so we have [x] separate contracts all very low value, it's about 1,000 screens in each, so you're talking maybe [x] £20,000 contracts or something. So it's a very bitty way of doing stuff. So we could still do it and we could pay for it, but in terms of the amount of paperwork or the amount of outcomes it becomes potentially unmanageable. [17685, PHE/NHSE, Area 2, Dec 2016]

This participant went on to indicate that he would prefer LAs to commission cervical screening as part of their sexual health contracts, but acknowledged the political difficulties for LAs to justify spending money on an area of service that was not formally their responsibility, especially given the context of diminishing LA budgets:

> In a way wouldn't it be so much easier if the local authorities just included it as part of their normal service? But their argument would be that's not our role, and how can we defend to the [elected] councillors that we're spending money on stuff that we don't have to, that someone else is meant to be spending money on? And our argument is well, it's just so much simpler and it's not a lot of money. That's the kind of discussion. And it eventually ends up with them withdrawing money and us saying well, we're not buying it either then.

Potential impacts on cervical screening rates

One LA commissioner suggested that the policy of his LA was to continue to facilitate opportunistic cervical screening tests at sexual health clinics, but not routine tests, because to provide the latter

For peer review only

would have a detrimental impact upon other sexual health services that the LA was now obligated to commission ("if we don't say no to [routine] smears, we'll be turning [other] people away, symptomatic patients away, or women needing contraception away. And that's our duty" [8384, LA, Area 1, Nov 2015]). He reported that local CCGs complained about this discontinuation of routine cervical screening at sexual health clinics, because there was insufficient capacity within general practice for CCGs to meet their cervical screening targets, and thus they required sexual health clinics to provide a proportion of cervical screening activity. One screening consultant developed this picture by suggesting that some localities would see a substantial reduction in screening activity because of a lack of capacity within primary care:

> …in some Local Authorities where the sexual health service is no longer doing cervical screening [it] will have a small impact but not a huge impact, in other areas, it will have a big impact on coverage, we'll see activity go down around it, because the workload is just going to come straight back to primary care, and in different areas primary care didn't realise this was happening, the recommissioning, hasn't got the capability and the capacity… [18352, PHE/NHSE, Area 1, Jan 2017]

Another screening consultant reflected that changes to NHSE footprints (i.e. the abolition of Area Teams and the new, more regional focus of the organisation) had implications for the provision of cervical screening:

> …say we wanted to sort out cervical screening coverage in GP practices, in [name of PCT] you've got [*x*] GP practices, bottom 20 per cent you could talk to the [*y*] practices. In my new patch we've got [many more than *x*] practices. So you have to think in a completely different way. [17685, NHSE, Area 2, Dec 2016]

Several participants from different localities in both of geographical areas pointed to long-standing challenges in ensuring good uptake rates for screening amongst their diverse local populations. There were concerns that these challenges would be exacerbated by a reduction in choice for women about where they could go for cervical screening tests:

> …you should have an integrated sexual health service where predominantly women can go in and get seen in one episode, in one place for all their sexual health needs, be that sexually transmitted infection testing and treatment and contraception. So I think probably in the past people worked very hard to get things like cervical screening into these services so that the needs of those women who perhaps wouldn't go to their local GP could be met in an environment they felt happy with. My feeling is now… that

For peer review only

perhaps the type of women who traditionally would have gone for cervical screening [at their sexual health clinic] might not feel so comfortable in that environment [of the GP practice]. So particularly, say, a lady from a South Asian background who goes to a single handed male GP with no practice nurse, that's the kind of traditional person who might have gone to a family planning clinic for their cervical screening. [9742, LA, Area 2, Jan 2016]

Quantitative findings

There were 14.12 million women eligible for screening in England in 2016 (NHS Digital 2016). Cervical screening rates decreased over time and the decline pre-dates the implementation of the HSCA in April 2013. Unassisted delivery rates also declined over time. The relative decline between the first year (2009/10) and the last year (2015/16) for unassisted deliveries (-4.17%) is larger than for cervical screening (-2.70%) (Table 3).

The changes in cervical screening rates over time were similar for practices in CCGs dealing with a single LA (-2.53%) compared with practices in CCGs working with multiple LAs (-2.87%). Figure 1 illustrates the trends in rates of cervical screening in the pre and post intervention periods for CCGs depending on the number of LAs they work with. There is a noticeable and sharp decline in the rates in both groups between 2011-12 and 2012-13.

For peer review only

Table 3: Numbers of general practices and mean rates of cervical screening and unassisted deliveries by year and by the number of LAs with which CCGs had to coordinate commissioning

Year	Number of general practices			Cervical screening rate (%)			Unassisted delivery rate (%)		
	1 LA	*2+ LAs*	*All*	*1 LA*	*2+ LAs*	*All*	*1 LA*	*2+ LAs*	*All*
2009/10	4,260	3,399	7,659	82.85	84.74	83.75	63.44	63.8	63.59
2010/11	4,261	3,403	7,664	82.74	84.61	83.63	63.19	63.76	63.44
2011/12	4,260	3,400	7,660	81.49	83.13	82.27	62.41	63.61	62.94
2012/13	4,249	3,399	7,648	81.28	82.87	82.04	62.17	63.08	62.57
2013/14	4,199	3,355	7,554	81.31	82.5	81.88	61.33	62.43	61.82
2014/15	4,125	3,302	7,427	81.21	82.54	81.85	60.67	61.98	61.25
2015/16	4,026	3,236	7,262	80.75	82.31	81.49	60.47	61.53	60.94
Relative Change between 2009/10 and 2015/16 (%)				*-2.53*	*-2.87*	*-2.70*	*-4.68*	*-3.56*	*-4.17*

Notes: *Mean cervical screening and unassisted delivery rates are weighted by the denominator.*

For peer review only

Figure 1: Uptake (%) of cervical screening pre and post HSCA

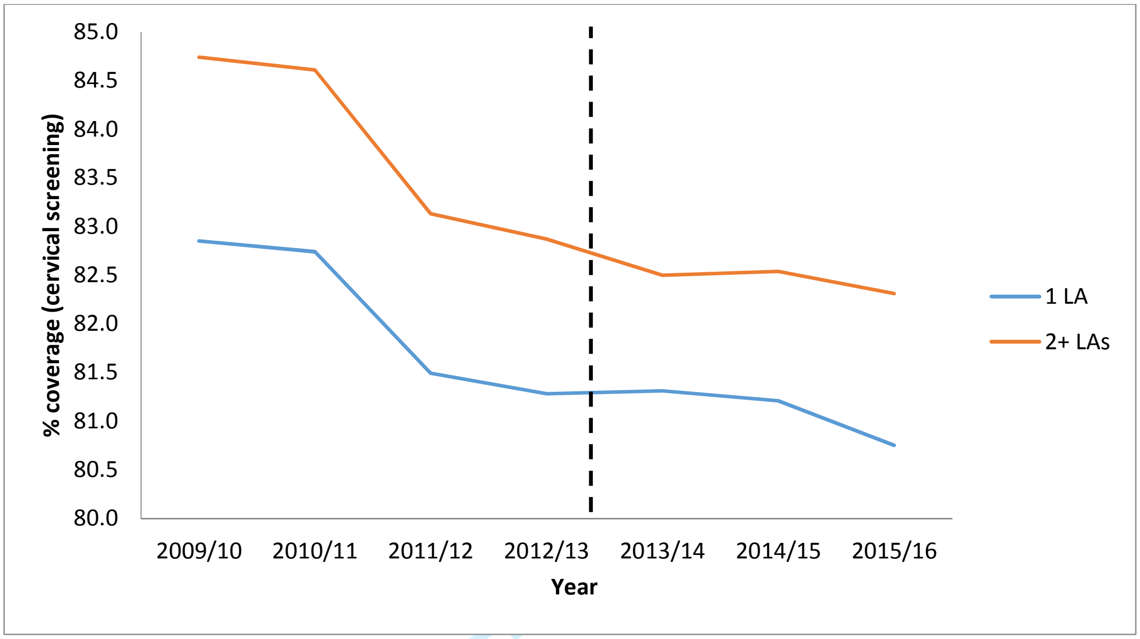

For peer review only

Comparing the averages for the pre-HSCA and post-HSCA years, cervical screening rates decreased by 0.39% more for GP practices located in CCGs working with multiple LAs compared to practices in CCGs working with a single LA. Unassisted birth rates decreased by 0.40% less for GP practices in CCGs working with multiple LAs compared to GP practices in CCGs working with a single LA. As maternity services were largely unaffected by the HSCA, we assume that these differential changes capture the unmeasured population influences that confound comparisons of the changes in the two groups of CCGs. Relative to the decreases in unassisted delivery rates, GP practices in CCGs working with multiple LAs experienced a decrease in cervical screening rates of 0.79% compared to practices in CCGs working with a single LA (Table 4).

The results are qualitatively similar for the triple difference models (for all years and 2011/12 onwards only) and for the lagged dependent variable estimator. The triple difference-in-differences estimates show that there was a differentially larger decline of 0.72% [95% confidence interval: -1.036 to -0.404] (Model 1) in cervical screening rates for practices located in CCGs working with more than one LA. The decrease is smaller over the shorter pre-period (0.327%; 95%CI -0.662 to 0.001, Model 2).

The direction of result is robust to the model specification and, although we rejected the assumption of parallel trends for Model 1 (all years), we could not reject the assumption for Model 2. We also found the same direction and a relatively similar size of result for Model 3 using the lagged dependent variable estimator, which yields unbiased estimates when pre-trends cannot be assumed to be parallel.

The results are also robust to different groupings of the number of LAs that CCGs work with. Table 5 includes model estimates comparing CCGs working with one or two LAs with CCGs working with more than two LAs. The direction of results is equivalent; and the scale and significance are either equivalent or increased. The same pattern is repeated in terms of tests of parallel trends. We cannot reject the null hypothesis of parallel trends for Model 2 and the LDV estimation is preferable to Model 1 in which we can reject the null hypothesis of parallel trends.

For peer review only

Table 4: Rates of cervical screening and unassisted birth for CCGs working with one and more than one LA, before and after the introduction of HSCA

	**CCGs working with one LA**			**CCGs working with more than one LA**				
**Condition**	Average in the Pre-HSCA years	Average in the Post-HSCA years	*Change*	Average in the Pre-HSCA years	Average in the Post-HSCA years	*Change*	**Difference-in-differences**	**Triple difference-in-differences**
	Percent							
**Affected (Cervical screening rates)**	82.09	81.09	*-1.00*	83.84	82.45	*-1.39*	**-0.39**	**-0.78**
**Unaffected (Unassisted birth rates)**	62.80	60.82	*-1.98*	63.56	61.98	*-1.58*	**0.40**	

Notes: values for pre and post are averages for all years in pre and post periods. The triple difference represents (the change over time in cervical screening rates for CCGs working with only one LA minus the change over time in cervical screening rates for CCGs working with more than one LA) minus (the change over time in unassisted birth rates for CCGs working with only one LA minus the change over time in unassisted birth rates for CCGs working with more than one LA). Differences are between raw figures.

For peer review only

Table 5: Triple difference-in-differences estimation†

	Difference-in-differences models				Lagged dependent variable models¤	
	**All years (1)**		**2011/12 onwards (2)**		**All years (3)**	
	Coefficient	*95% CI*	*Coefficient*	*95% CI*	*Coefficient*	*95% CI*
**Triple difference-in-differences‡**	-0.720***	[-1.036; -0.404]	-0.327*	[-0.662; 0.001]	-0.502***	[-0.757; -0.247]
**Triple difference-in-differences‡ (Alternative grouping of CCGs®)**	-0.889***	[-1.236; -0.542]	-0.515***	[-0.882; -0.148]	-0.529***	[-0.808; -0.251]
**Number of observations**	105,745		75,099		44,472	
**Test of parallel trends**	**F (3, 7672)**	**p-value**	**F (1, 7667)**	**p-value**	**N/A**	
	6.87	0.001	9.4	0.3316		

*Notes: ***indicates p < 0.01; *indicates p < 0.1.*

†*Values are regression estimations from weighted OLS models including practice-condition specific fixed effects, full interaction of year with condition; and full interaction of year with the dummy for (N of LAs). Robust standard errors. Weighted by denominator. Clustered by practice.*

‡*The triple difference represents (the change over time in cervical screening rates for CCGs working with only one LA minus the change over time in cervical screening rates for CCGs working with more than one LA) minus (the change over time in unassisted birth rates for CCGs working with only one LA minus the change over time in unassisted birth rates for CCGs working with more than one LA).*

For peer review only

¤ *LDV also contains values of dependent variable in each of the pre intervention years. Estimated only on post intervention years.*

® *This model uses an alternative grouping of CCGs based on the number of LAs they work with [(1 or 2) vs. (more than 2)]*

For peer review only

DISCUSSION

We conducted a mixed methods study exploring the impact of changes associated with the HSCA in the English NHS on cervical screening rates. We carried out qualitative interviews with senior figures from a variety of relevant organisations in two large, socioeconomically diverse areas of England. Analysis of these interviews suggested that cervical screening commissioning had become more complex, with responsibilities between organisations less certain, as a consequence of the HSCA. Some interviewees predicted that there would be a detectable reduction in cervical screening rates, and that this reduction would be concentrated in particular areas. We subsequently sought to test this prediction via a difference-in-differences analysis of publically-available data for cervical screening activity. To control for unmeasured confounders, we compared with trends in unassisted birth rates because the commissioning of maternity services was unchanged pre and post HSCA.

Interviewees suggested a number of factors that might contribute to a reduction in cervical screening activity. Sexual health service commissioning responsibility now lies with LAs while NHS England is responsible for commissioning national screening services, which includes cervical screening. In a context of austerity and cuts to LA budgets, many LAs were retendering their sexual health services and not including routine cervical screening as part of the bundle of service which they commission from sexual health providers. NHS England is also seemingly reluctant to commission sexual health clinics to perform cervical screening tests because this would entail a multitude of low-value contracts with numerous providers. This would be administratively laborious and practically difficult given the large size of the administrative areas they operate on and the relatively low levels of staff they have attending to these areas.

As a result, there were predictions that cervical screening activity might decline in some areas, either because of a lack of capacity in primary care (which had previously been relied upon the additional capacity in sexual health clinics), or because there was now a lack of choice for women who may have preferred to attend sexual health clinics rather than their GP practice. The quantitative analysis supports these interview findings. GP practices located in CCGs dealing with multiple LAs (and therefore most exposed to potential disruption in services) experienced a larger decrease over time in cervical screening rates compared with practices in CCGs dealing with a single LA. The opposite pattern was observed for unassisted births, which decreased more over time in the CCGs dealing with a single LA. The triple difference-in-differences analyses confirmed that the effects were statistically significant and robust to different model specifications.

For peer review only

Taken together, our findings suggest that there is an urgent need for clarification as to who holds the budget, and therefore who should be commissioning, cervical screening in the English NHS, and for local agreements to ensure that issues over funding and budgets do not disrupt screening programmes. Research is also needed to explore where women would prefer to have their cervical screening tests, and for services to be developed to meet these preferences.

Potential confounders and study strengths

We took 2009 as our starting point for pre-HSCA cervical screening activity. Two potential confounders to our results were considered. First, the high-profile case of Jade Goody, a 27-year-old reality TV star who was diagnosed with cervical cancer in August 2008 and died in March 2009. The contemporaneous media attention and publicity has been linked with a substantial increase in cervical screening rates (around half a million) during the time of Goody's diagnosis and death. Previous impacts of high-profile cases of celebrity cancer diagnoses upon population behaviour have tended to be brief and immediate rather than longer-lasting, and therefore we are confident that from 2010 onwards, rates of cervical screening returned towards underlying trends (Lancucki et al. 2012). Second, the UK's Human Papillomavirus (HPV) vaccination programme was introduced in 2008 in order to reduce the incidence of cervical cancer (Public Health England 2015). The vaccine is offered to all girls aged 12-13, and figures for 2008-2014 show high uptake rates of just under 90%. It is likely that this vaccination programme will contribute to a reduction in cervical screening activity. However, the first cohort of women in the programme, i.e. those aged 12-13 in 2008, were around 21-22 years old in 2016/17, and hence at the time of our study were too young to have been invited for routine cervical screening (which begins at age 25). We can therefore be confident that any changes to cervical screening rates cannot yet be attributed directly to the HPV programme, but any future research into cervical screening rates needs to account for this programme.

We considered whether the results were sensitive to the groups that CCGs were assigned to on the basis of the number of LAs they work with. The direction of results was equal, and the strength and significance was increased when comparing CCGs working with one or two LAs with those CCGs working with more than two LAs. We also considered whether the results were sensitive to the choice of comparison indicator (unassisted births) for maternity services. We tested whether the results would hold for another indicator of maternal outcome: the rate of deliveries by caesarean section. We observed the same direction and significance of results for this indicator as well.

A notable strength of this study is that it was part of a larger, longitudinal study of major healthcare system reform conducted by a multidisciplinary research team. This facilitated the development of the novel, iterative, exploratory mixed methods approach presented here. This study shows the

For peer review only

benefits of designing a mixed method study which allows a qualitative analysis to feed into the design of a subsequent quantitative analysis to test the claims being made in interviews among participants. There is a cumulative and reinforcing effect in this which provides a strong indication that the HSCA did have a negative effect on cervical screening rates especially in places more disrupted by the reforms.

ACKNOWLEDGMENTS

The authors would like to acknowledge the valuable advice of the Project Advisory Group.

For peer review only

REFERENCES

Barbour, R. (1999). The case for combining qualitative and quantitative approaches in health services research. *Journal of Health Services Research & Policy*, 4(1), pp.39–43.

Best, A. et al. (2012). Large-system transformation in health care: a realist review. *Milbank Quarterly*, 90(3), pp.421–456.

Braun, V. and Clarke, V. (2006). Using thematic analysis in psychology. *Qualitative Research in Psychology*, 3(2), pp.77–101.

Checkland, K. et al. (2016). Complexity in the new NHS: longitudinal case studies of CCGs in England. *BMJ Open*, 6(1), p.e010199.

Department of Health. (2010). *Equity and excellence: liberating the NHS*. London: The Stationery Office. [online]. Available from: http://www.dh.gov.uk/en/Publicationsandstatistics/Publications/PublicationsPolicyAndGuidance/DH_117353 [Accessed March 21, 2011].

Department of Health. (2000). *The NHS Plan: a plan for investment, a plan for reform*. London: The Stationery Office. [online]. Available from: http://www.dh.gov.uk/en/Publicationsandstatistics/Publications/PublicationsPolicyAndGuidance/DH_4002960 [Accessed August 31, 2012].

Edwards, N. (2010). *The triumph of hope over experience: Lessons from the history of reorganisation*. NHS Confederation.

Guest, G. (2013). Describing Mixed Methods Research: An Alternative to Typologies. *Journal of Mixed Methods Research*, 7(2), pp.141–151.

Ham, C. et al. (2015). *The NHS under the coalition government. Part one: NHS reform*. London. [online]. Available from: http://www.kingsfund.org.uk/publications/nhs-under-coalition-government [Accessed November 18, 2015].

Health and Social Care Act. (2012). *(c.7)*. London: HMSO.

Imbens, G.W. and Wooldridge, J.M. (2009). Recent developments in the econometrics of program evaluation. *Journal of Economic Literature*, 47(1), pp.5–86.

Lale, A.S. and Temple, J.M.F. (2016). Has NHS reorganisation saved lives? A CuSum study using 65 years of data. *Journal of the Royal Society of Medicine*, 109(1), pp.18–26.

Lancucki, L. et al. (2012). The impact of Jade Goody's diagnosis and death on the NHS Cervical Screening Programme. *Journal of Medical Screening*, 19(2), pp.89–93.

NHS Digital. (2016). *Cervical Screening Programme, England – 2015-16: report*. [online]. Available from: https://files.digital.nhs.uk/publicationimport/pub22xxx/pub22414/nhs-cerv-scre-prog-eng-2015-16-rep.pdf [Accessed April 4, 2017].

NHS England and Public Health England. (2013). *Immunisation and screening national delivery framework & local operating model*. [online]. Available from: https://www.england.nhs.uk/wp-content/uploads/2013/05/del-frame-local-op-model-130524.pdf.

For peer review only

O'Neill, S. et al. (2016). Estimating causal effects: considering three alternatives to difference-in-differences estimation. Health Services and Outcomes Research Methodology, 16(1–2), pp.1–21.

Peckham, S. et al. (2015). *The organisation and delivery of health improvement in general practice and primary care: a scoping study*. National Institute for Health Research.

Peckham, S. et al. (2017). Views of public health leaders in English local authorities – changing perspectives following the transfer of responsibilities from the National Health Service to local government. *Local Government Studies*, 43(5), pp.842–863.

Propper, C. (1996). Market structure and prices: The responses of hospitals in the UK National Health Service to competition. *Journal of Public Economics*, 61(3), pp.307–335.

Public Health England. (2015). *Human Papillomavirus (HPV) Vaccine Coverage in England, 2008/09 to 2013/14*.

Weiss, C.H. (1998). *Evaluation: methods for studying programs and policies*. New Jersey: Prentice Hall.

For peer review only

FOOTNOTES

Author statement / Contributors: KC designed the study with input from MS, NM, PA, AC. JH, AH, LWG gathered and analysed the qualitative data. TM and MS designed the quantitative evaluation and conducted this analysis. JH drafted the manuscript to which all authors made substantial contributions. All authors approved the final version and agree to be accountable for all aspects of the analysis.

Funding: The report is based on independent research commissioned and funded by the NIHR Policy Research Programme ('Understanding the new commissioning system in England: contexts, mechanisms and outcomes', PR-R6-1113-25001). The views expressed in the publication are those of the author(s) and not necessarily those of the NHS, the NIHR, the Department of Health, arm's-length bodies or other government departments.

Competing interests: None declared.

Patient consent: Not required.

Data sharing statement: No additional data are available.

BMJ Open

Exploring the impacts of the 2012 Health and Social Care Act reforms to commissioning upon clinical activity in the English NHS: A mixed methods study of cervical screening

Journal:	*BMJ Open*
Manuscript ID	bmjopen-2018-024156.R1
Article Type:	Research
Date Submitted by the Author:	11-Dec-2018

Complete List of Authors:	Hammond, Jonathan; University of Manchester, Division of Population Health, Health Services Research, and Primary Care; School of Health Sciences; Faculty of Biology, Medicine and Health<br>Mason, Thomas; University of Manchester, Manchester Centre for Health Economics; Division of Population Health, Health Services Research, and Primary Care; School of Health Sciences; Faculty of Biology, Medicine and Health<br>Sutton, Matt; University of Manchester, Division of Population Health, Health Services Research, and Primary Care; School of Health Sciences; Faculty of Biology, Medicine and Health<br>Hall, Alex; University of Manchester, Division of Nursing, Midwifery & Social Work; School of Health Sciences<br>Mays, Nicholas; London School of Hygiene and Tropical Medicine, Public Health and Policy<br>Coleman, Anna; University of Manchester, Division of Population Health, Health Services Research, and Primary Care; School of Health Sciences; Faculty of Biology, Medicine and Health<br>Allen, Pauline; Health Services Research Unit<br>Warwick-Giles, Lynsey; University of Manchester, Division of Population Health, Health Services Research, and Primary Care; School of Health Sciences; Faculty of Biology, Medicine and Health<br>Checkland, Kath; University of Manchester, Division of Population Health, Health Services Research, and Primary Care; School of Health Sciences; Faculty of Biology, Medicine and Health

**Primary Subject Heading**:	Health policy
Secondary Subject Heading:	Sexual health, Health services research, Public health, Health economics
Keywords:	NHS, Health policy < HEALTH SERVICES ADMINISTRATION & MANAGEMENT, Health system reform, Screening, Mixed methods, Commissioning

SCHOLARONE™
Manuscripts

Exploring the impacts of the 2012 Health and Social Care Act reforms to commissioning upon clinical activity in the English NHS: A mixed methods study of cervical screening

Authors

- *Dr Jonathan Hammond [Corresponding author]*

Division of Population Health, Health Services Research, and Primary Care; School of Health Sciences; Faculty of Biology, Medicine and Health; Williamson Building (6th floor), The University of Manchester, Manchester, M13 9PL, UK
Email: jonathan.hammond@manchester.ac.uk; Tel.: 0161 2757648

- *Mr Thomas Mason*

Manchester Centre for Health Economics; Division of Population Health, Health Services Research, and Primary Care; School of Health Sciences; Faculty of Biology, Medicine and Health; The University of Manchester, Manchester, UK

- *Prof Matt Sutton*

Division of Population Health, Health Services Research, and Primary Care; School of Health Sciences; Faculty of Biology, Medicine and Health; The University of Manchester, Manchester, UK

- *Dr Alex Hall*

Division of Nursing, Midwifery and Social Work; School of Health Sciences; The University of Manchester, Manchester, UK

- *Prof Nicholas Mays*

Department of Health Services Research and Policy; Faculty of Public Health and Policy; London School of Hygiene and Tropical Medicine, London, UK

- *Dr Anna Coleman*

Division of Population Health, Health Services Research, and Primary Care; School of Health Sciences; Faculty of Biology, Medicine and Health; The University of Manchester, Manchester, UK

- *Prof Pauline Allen*

Department of Health Services Research and Policy; Faculty of Public Health and Policy; London School of Hygiene and Tropical Medicine, London, UK

- *Dr Lynsey Warwick-Giles*

Division of Population Health, Health Services Research, and Primary Care; School of Health Sciences; Faculty of Biology, Medicine and Health; The University of Manchester, Manchester, UK

- *Prof Kath Checkland*

Division of Population Health, Health Services Research, and Primary Care; School of Health Sciences; Faculty of Biology, Medicine and Health; The University of Manchester, Manchester, UK

For peer review only - http://bmjopen.bmj.com/site/about/guidelines.xhtml

ABSTRACT

Objectives: Explore the impact of changes to commissioning introduced in England by the Health and Social Care Act 2012 (HSCA) on cervical screening activity in areas identified empirically as particularly affected organisationally by the reforms.

Methods: Qualitative followed by quantitative methods. Qualitative: semi-structured interviews (with NHS commissioners, managers, clinicians, senior administrative staff from Clinical Commissioning Groups (CCGs), local authorities, service providers), observations of commissioning meetings in two metropolitan areas of England. Quantitative: triple-difference analysis of national administrative data. Variability in the expected effects of HSCA on commissioning was measured by comparing CCGs working with one local authority with CCGs working with multiple local authorities. To control for unmeasured confounders, differential changes over time in cervical screening rates (amongst women, 25-64 years) between CCGs more and less likely to have been affected by HSCA commissioning organisational change were compared with another outcome – unassisted birth rates – largely unaffected by HSCA changes.

Results: Interviewees identified that cervical screening commissioning and provision was more complex and 'fragmented', with responsibilities less certain, following the HSCA. Interviewees predicted this would reduce cervical screening rates in some areas more than others. Quantitative findings supported these predictions. Areas where CCGs dealt with multiple local authorities experienced a larger decline in cervical screening rates (1.4 percent) than those dealing with one local authority (1.0 percent). Over the same period, unassisted deliveries decreased by 1.6 percent and 2.0 percent, respectively, in the two groups.

Conclusions: Arrangements for commissioning and delivering cervical screening were disrupted and made more complex by the HSCA. Areas most affected saw a greater decline in screening rates than others. The fact that this was identified qualitatively and then confirmed quantitatively using strengthens this finding. The study suggests large scale health system reforms may have unintended consequences, and that complex commissioning arrangements may be problematic.

Keywords:

Screening; Health policy; Mixed methods; Commissioning; NHS; Health system reform

For peer review only - http://bmjopen.bmj.com/site/about/guidelines.xhtml

ARTICLE SUMMARY

Strengths and limitations of this study:

- Few studies have investigated in detail the impacts of large-scale health system change.
- This study combines detailed qualitative data exploring impacts on the system with quantitative exploration of important outcomes, supporting causal inference.
- Based on qualitative findings, we developed a quantitative measure for assessing the extent of disruption to the English NHS commissioning system as a result of the 2012 Health and Social Care Act.
- We found that cervical screening rates decreased more post-Act in areas that had experienced higher levels of disruption.

INTRODUCTION

Structural re-organisations of publicly financed health care systems, driven by central government or other state agencies, are frequently employed with the objective of improving health care delivery, and thus population health outcomes, whilst reducing or containing costs.[1] However, such endeavours can be disruptive and expensive.[2] It is important to understand what possible impacts these reorganisations have in order to understand their value.[3]

In the English NHS, attempts to evaluate the impact of reorganisations have typically used operational indicators (e.g. bed availability, numbers of staff) and measures of clinical activity because their improvement was the stated goal of government policy (e.g. The NHS Plan,[4]). Other studies have attempted to assess the impacts of reforms by measuring their effects on prices, quality and quantity of provision.[5] Most studies have relied upon quantitative analysis of measures that were explicitly targeted by policy reforms. There is a need for approaches which combine qualitative and quantitative methods to generate a deeper understanding of the impacts of structural reorganisation.[6]

The most recent structural reorganisation of the English NHS, the Health and Social Care Act[7] (hereafter 'HSCA' or 'the Act'), was introduced in April 2013 and included wide-ranging changes to the health services commissioning system. We explore whether changes to the commissioning of cervical screening services resulting from the Act affected uptake. This analysis uses a relatively novel mixed methods approach. An initial 'bottom-up' qualitative analysis allowed us to identify problematic issues associated with the HSCA for those working locally in the health service

For peer review only - http://bmjopen.bmj.com/site/about/guidelines.xhtml

commissioning system. This process highlighted the disruption to established commissioning arrangements and cervical screening as a clinical activity which may be specifically affected by this disruption. We then developed a quantitative investigation to explore this more fully. Together these analyses allow us to infer causation.

The HSCA and changes to cervical screening commissioning

The HSCA is regarded as one of the most wide-ranging legislative reforms in the history of the English NHS.[8] Primary Care Trusts (PCTs), 152 organisations previously responsible for the commissioning of primary, community and secondary health services from providers on behalf of local populations, were abolished. Their commissioning functions were split between three groups of organisations: 211 (now 195) newly created Clinical Commissioning Groups (CCGs), membership organisations constituted by GP (family doctor) practices, given responsibility for commissioning services for their local populations; NHS England, a new arm's-length governmental body with responsibility for authorising and assessing CCGs, and commissioning some services at a national level; and top-tier and single-tier elected local authorities, which took responsibility for the majority of public health services for the first time since 1974. In addition, Public Health England (PHE) was created as an executive agency of the Department of Health, to unify the diverse public health profession and provide expert support for local public health services.

In some service areas, the transfer of commissioning responsibilities was relatively straightforward (e.g. the commissioning of routine orthopaedic surgery was passed from PCTs to CCGs with minimal alteration to the bundle of services involved). In other service areas, the transfers were much more complex, particularly the commissioning of national screening programmes and sexual health services, as a result of changes to public health commissioning. Pre-HSCA, national screening programmes and sexual health services were both commissioned by PCTs. Cervical screening was largely provided by GP practices, which received additional funding linked to levels of activity,[9] but some women opted to have their cervical smears in PCT-commissioned sexual health clinics. Post-HSCA, responsibility for public health services, including most sexual health services, was transferred to local authorities. The underlying programme theory (i.e. the explicit expectation about how the policy would work;[10]) was that local authorities would be better placed to address the wider determinants of health and wellbeing than the NHS because they could link public health services with their existing responsibilities, such as for transport and housing.[11] NHS England took responsibility for commissioning national screening programmes.[8] NHS England's regional teams are responsible for commissioning screening programmes, supported by PHE staff 'embedded' within NHSE's screening and immunisation teams.[12] There was no identifiable underlying

For peer review only - http://bmjopen.bmj.com/site/about/guidelines.xhtml

programme theory for this specific change to screening programme commissioning. However, it is notable that, in contrast to the emphasis placed upon localism associated with the creation of CCGs and with the transfer of public health to local authorities, screening commissioning became more centralised as a consequence of the HSCA.

Table 1 shows the organisations with responsibilities of relevance to the commissioning of sexual health, including cervical cancer screening services, pre and post-HSCA. It illustrates how responsibility for such services, previously commissioned by PCTs, was split between different agencies. This increased complexity and fragmentation of responsibilities had the potential to disrupt service commissioning.[13]

For peer review only

For peer review only - http://bmjopen.bmj.com/site/about/guidelines.xhtml

Table 1: Organisations of significance to the commissioning of cervical screening pre and post HSCA

Pre-HSCA	
Primary Care Trusts	Responsible for all public health commissioning, including sexual health services; Responsible for commissioning national screening programmes, including cervical screening
UK National Screening Committee	Provision of advice and support to NHS organisations on population screening programmes
Post-HSCA	
Local authorities	Responsible for most public health commissioning, including most sexual health services (contraception over and above GP contract, testing and treatment of sexually transmitted infections, sexual health advice and promotion)
NHS England (NHSE)	Responsible for commissioning national screening programmes, including cervical screening, and some sexual health services (notably contraception through GP contract, and HIV treatment)
Public Health England (PHE) (including National Screening Committee)	Hosts UK National Screening Committee secretariat, which retains the same role; PHE staff embedded in sub-regional NHSE screening and immunisation teams to provide expertise and 'leadership'
Clinical Commissioning Groups (CCGs)	Responsible for improving clinical outcomes for their patients and prevent premature death (as part of the NHS Outcomes Framework, which CCGs are held to account on by NHSE); GP members are funded to carry out cervical screening through the GP Contract

This analysis comes from a study designed to foster emergent interplay between qualitative and quantitative data analysis.[14,15] The focus on cervical screening was not established at the project design stage but driven by the initial qualitative interview findings related to sexual health commissioning arrangements and screening activity post-HSCA. These findings prompted us to consider a quantitative exploration of predictions made by interviewees relating to potential changes in cervical screening activity.

METHODS

Study context and design

This analysis forms part of a longitudinal project, with data collected between January 2015 and December 2017, into the effect of the HSCA upon the commissioning system in England. We

For peer review only - http://bmjopen.bmj.com/site/about/guidelines.xhtml

combined a qualitative and quantitative exploration of the commissioning of services in two large, socioeconomically diverse metropolitan areas of England with a national level quantitative study of commissioning outcomes. We used a sequential mixed methods approach in which initial qualitative data collection and analysis were used to shape an ensuing quantitative analysis using routinely available data. We therefore present the qualitative and quantitative methods and findings in the order undertaken, and integrate them in the Discussion.

Patient and public involvement

Our interest in exploring the impact of systemic commissioning change on cervical screening rates was driven initially by concerns expressed by interviewees about potentially negative consequences for patients relating to new arrangements. Patients were not directly involved in the design of, or recruitment to, the overarching project, but an advisory group including a patient representative met regularly throughout the project and played an important role in supporting its development. We presented our initial qualitative findings relating to cervical screening, and early ideas for developing a mixed-methods investigation, to our advisory group and were encouraged by our patient representative to pursue this. The results of the broader project were disseminated to participants, and the advisory group, in the form of a series of short reports focusing on specific areas of commissioning and the final report.

Qualitative component

Setting, participants, sampling and data collection

The qualitative component took place between March 2015 and August 2017, focussing on two metropolitan 'health economies' covering a geographical population and a group of commissioning organisations and providers with close operational links. Across both areas, we conducted 143 interviews (each typically an hour in length), 93 of which related to sexual health commissioning, with clinical and non-clinical commisioners, managers, clinicians, and senior administrative staff from CCGs, local authorities, service providers and third sector organisations. Organisations and participants were sampled purposively for variation in type and role. We identified participants through organisational websites, personal contacts and through 'snowballing' in which we asked participants to recommend other potential participants. Additionaly, eight hours of meetings of an inter-organisaional sexual health co-ordinating group involving sexual health commissioners and providers were observed in one of the areas.

Interviews focused upon the commissioning system pre- and post-HSCA, exploring continuities and changes to personal and organisational roles, key issues and challenges, accountability and

For peer review only - http://bmjopen.bmj.com/site/about/guidelines.xhtml

performance management, interorganisational relationships and communication, and commissioning decision-making. Interviews took place either in person (usually in participants' offices) or over the telephone. All interviews were audio-recorded and all interviewees were provided with written information about the study before consenting to participate.

Data analysis

Audio recordings of interviews were transribed verbatim, and observational notes from meetings were produced contemporaneously. Transcripts and observational notes were imported into NVivo 10 software and analysed thematicly by JH and AH.[16] This involved repeated readings of transcripts to become sufficiently familiar with their contents, identifying initial codes and coding chunks of data, searching for themes and then iteratively defining and re-constituting themes. Our findings (see below) contained some predictions made by participants regarding changes in cervical screening activity as a consequence of the Act. This prompted us to explore these predictions in a quantitative analysis, which we now describe.

Quantitative component

Interviewees identified that the HSCA had introduced confusion over responsibility for the commissioning of cervical screening services, and had increased variability of provision. They hypothesised that cervical screening rates might be reduced by the new commissioning arrangements; this prompted discussions amongst the research team and advisory group about developing a way of testing these predictions quantitatively as far as routine data would permit. As the Act had been implemented in all areas simultaneously removing the scope for a quasi-experimental approach, we sought to identify a measure of variability in the extent to which the Act would have been expected to make commissioning more difficult in each area. One of the features of the post-HSCA system was that some, but not all, CCGs were established which crossed local authority boundaries. Some CCGs related to as many as three separate local authorities. Local authorities were now directly involved in sexual health services' commissioning. Findings revealed that CCGs experienced extra challenges when they had to engage with more than one local authority. This suggested that the burden of additional inter-organisational coordination might have consequences for commissioning.

As each local authority developed its own approach to cervical screening in its local sexual health clinics, we explored the possibility that GP practices located in CCGs which had to work with more than one local authority might experience lower screening rates compared with practices located in CCGs which had only to deal with one local authority. We compare the demographic characteristics

For peer review only - http://bmjopen.bmj.com/site/about/guidelines.xhtml

of these two groups in Table 2. The 89 CCGs dealing with more than one local authority had a slightly older population profile than the 119 CCGs which dealt with only one local authority but were otherwise highly comparable.

Table 2: CCG demographic characteristics depending on the number of local authorities that the CCG needs to work with

	One local authority	More than one local authority
Number of CCGs	119	89
Population (millions)	29.5	26.9
Female	50.0%	50.5%
Aged 0-9	12.3%	11.4%
Aged 10-19	11.2%	11.2%
Aged 20-39	30.0%	24.8%
Aged 40-59	26.5%	27.7%
Aged 60-79	16.0%	19.7%
Aged 80 and over	4.1%	5.3%

For peer review only

Because cervical screening rates may be influenced by other factors that we cannot observe and may change over time in different ways between the two groups of CCGs, we also compared screening rates with an outcome that was unlikely to have been affected by the introduction of HSCA. We used unassisted births (i.e. uncomplicated deliveries which did not require any intervention) as a percentage of all maternal deliveries, since the commissioning of maternity services was largely unchanged by the Act.

We applied a triple-difference approach. The triple difference represents (the change over time in cervical screening rates for CCGs working with only one local authority minus the change over time in cervical screening rates for CCGs working with more than one local authority) minus (the change over time in unassisted birth rates for CCGs working with only one local authority minus the change over time in unassisted birth rates for CCGs working with more than one local authority).

The screening rate is defined as the percentage of women aged between 25 and 64 years who had received a cervical screening test in the preceding five years. This indicator was derived from annual,

For peer review only - http://bmjopen.bmj.com/site/about/guidelines.xhtml

practice-level data from the Quality and Outcomes Framework (QOF), 2009-10 to 2015-16. The comparison indicator is unassisted births as a percentage of all maternal deliveries. This indicator was produced using operation codes in Hospital Episode Statistics (HES) for 2009-10 to 2015-16. We aggregated the spell-level data by general practice and financial year.

The key assumption underpinning the triple-difference estimator is that, conditional on the other variables in the model, the differences in the changes over time in the intervention indicator (cervical screening) between the 'exposed' and the 'control' areas (in this case, CCGs working with one local authority versus CCGs working with more than one) would have been the same as the differences in the changes over time in the comparison indicator (unassisted births) between the exposed and control areas in the absence of the intervention. This is a more complex version of the 'parallel trends' assumption required for the double-difference, or difference-in-differences, estimator.[17]

A popular test of this assumption in the double-difference case is that there are parallel trends over time in the outcomes in the intervention and comparison group in the pre-intervention period. For our triple-difference case, we used an F-test to assess the joint significance of interactions between the year effects and the binary variable representing the combination of exposed area and treated indicator in the pre-period.

We also used the lagged dependent variable (LDV) estimator. This model is estimated only on data in the post-intervention period and is a less biased estimator of treatment effects when the assumption of parallel pre-trends does not hold.[18] We set up the LDV model to generate the equivalent impact estimate as the triple-difference model. The model included: dummy variables for year; values of the dependent variable in each of the pre-intervention periods; a dummy variable classifying practices depending on whether they were located in CCGs working with more than one local authority; interactions between year and condition dummies; interactions between values of the dependent variable in the pre-intervention period and the condition dummy; and an interaction between the dummy variable classifying practices depending on whether they were located in CCGs working with more than one local authority and the condition dummy. The final term is the impact estimate, showing whether cervical screening was differentially affected after the introduction of the reforms for local authorities working with multiple CCGs. We estimated the regression models in Stata 14.1 using dummy variable weighted least squares regression with fixed effects for practice-indicator combinations. The ways in which these models are estimated using regression analyses are described formally in the technical appendix. As the dependent variable is a proportion, and constrained to lie between 0 and 1, we used the empirical logit transformation and back-

For peer review only - http://bmjopen.bmj.com/site/about/guidelines.xhtml

transformed the coefficients and associated 95% confidence intervals using the mean value of the cervical screening rate.[19] We clustered the standard errors at the GP practice level.[20]

RESULTS

Qualitative findings

Interviewees told us that CCGs working with more than one local authority experienced a number of challenges, including: finding sufficient capacity to engage in multiple meetings of the same type with different local authorities; managing additional collaborative relationships; working with organisations experiencing different financial pressures from each other with different approaches to public health spending; and attempting to develop integrated health and social care arrangements with one local authority that did not have unintended and undesirable consequences for plans with another. The following extract illustrates issues relating to difficulties commissioning a single service offer for CCG patients and the additional resources required for a CCG working with multiple local authorities. (Interview data extracts are denoted by square brackets with numerical participant ID, participant's organisation type, Area (1 or 2), and month and year of the interview.)

> We do have two sets of safeguarding arrangements. So I guess at one level, one can say there is a risk of and there are examples of services being subtly different. Equally, you've got to service two times the number of these processes, which can be quite labour-intensive. [2778, CCG, Area 1, April 2015]

In our analysis relating directly to issues surrounding the commissioning and provision of cervical screening post-HSCA, we identified two main themes: confusion and uncertainty regarding budgets and responsibilities, and potential impacts on cervical screening rates. Many of the issues discussed below are likely to be exacerbated when the number of interacting commissioning organisations in a local area are increased.

Confusion and uncertainty regarding budgets and responsibilities

Before the HSCA, both cervical screening and sexual health services were commissioned by PCTs. As one screening and immunisation lead outlined, cervical screening tests (sometimes referred to as smear tests) were provided by GP practices, but patients could usually also have them at sexual health clinics [17685, NHSE, Area 2, Dec 2016]. Whereas pre-HSCA PCTs held the budget for both cervical screening and sexual health services, following the Act these budgets were separated. This meant that the local authority budget and responsibility for sexual health did not extend to cervical screening. One local authority public health consultant reported that, in spite of this, PHE was

For peer review only - http://bmjopen.bmj.com/site/about/guidelines.xhtml

sending letters to patients explicitly stating that they could choose to attend either their GP practice or their local sexual health clinic for their cervical screening test. This highlights confusion regarding commissioning arrangements and budgetary responsibility:

> Public Health England were writing around to people saying …you're due your smear, you can go to your general practice or you can go to your local sexual health clinic. And we said, but we don't have the money for them to do that, they can't come here routinely unless you're going to pay us for that. Public Health England, the screening people, they have the money to pay for the smears. But in all the moving around of the budgets, the money for smears that were taken outside general practice doesn't seem to be anywhere [8384, local authority, Area 1, Nov 2015].

One participant from NHS England offered a different perspective. He argued that the public health budget of each local authority reflected the levels of cervical screening activity that had taken place in its sexual health clinics pre-HSCA. However, this is not clear because, in the past, the funding was not "disaggregated" [4058, NHSE, Area 1, Jun 2015]. Therefore, it is not possible to establish what the pre-HSCA sexual health component of the public health budget covered.

> …they [local authorities] think they're not being paid for it [cervical screening]. But, actually, in truth, whatever they were doing at the point of transition if they were doing loads of cervical smears they were just doing loads of cervical smears, so they had the money. There wasn't a problem when they were doing them before, it's just the money wasn't disaggregated. However local authorities have been put under significant pressure in their public health teams to reduce their budgets. So these kinds of things are examples where you can say it's not our responsibility so therefore we're taking that element out [4058, NHSE, Area 1, Jun 2015].

The above quote illustrates a phenomenon reported by a number of participants that local authorities had re-procured their sexual health services and had taken a position that they would not commission their sexual health provider(s) to do routine cervical screening, because it was not their commissioning responsibility. However, as one member of a screening team in Area 2 illustrated, NHSE was also reluctant to explicitly commission sexual health services to provide cervical screening, seemingly because of administrative challenges relating to numerous low-value contracts with providers:

> So cervical screening, we could go to every sexual health provider and have a separate contract. The difficulty again becomes around commissioning capacity. So, I think we've

For peer review only - http://bmjopen.bmj.com/site/about/guidelines.xhtml

got [x] local authorities, so we have [x] separate contracts all very low value, it's about 1,000 screens in each, so you're talking maybe [x] £20,000 contracts or something. So, it's a very bitty way of doing stuff. So, we could still do it and we could pay for it, but in terms of the amount of paperwork or the amount of outcomes it becomes potentially unmanageable. [17685, PHE/NHSE, Area 2, Dec 2016]

This participant went on to indicate that he would prefer local authorities to commission cervical screening as part of their sexual health contracts, but acknowledged the political difficulties for local authorities to justify spending money on an area of service that was not formally their responsibility, especially given the context of diminishing local authority budgets:

In a way, wouldn't it be so much easier if the local authorities just included it as part of their normal service? But their argument would be that's not our role, and how can we defend to the [elected] councillors that we're spending money on stuff that we don't have to, that someone else is meant to be spending money on? And our argument is well, it's just so much simpler and it's not a lot of money. That's the kind of discussion. And it eventually ends up with them withdrawing money and us saying well, we're not buying it either then. [17685, PHE/NHSE, Area 2, Dec 2016]

Potential impacts on cervical screening rates

One local authority commissioner suggested that the policy of his local authority was to continue to facilitate opportunistic cervical screening tests at sexual health clinics, but not routine tests, because to provide the latter would have a detrimental impact upon other sexual health services that the local authority was now obligated to commission ("if we don't say no to [routine] smears, we'll be turning [other] people away, symptomatic patients away, or women needing contraception away. And that's our duty" [8384, local authority, Area 1, Nov 2015]). He reported that local CCGs complained about this discontinuation of routine cervical screening at sexual health clinics, because there was insufficient capacity within general practice for CCGs to meet their cervical screening targets, and thus they required sexual health clinics to provide a proportion of cervical screening activity. One screening consultant developed this point by suggesting that some localities would see a substantial reduction in screening activity because of a lack of capacity within primary care:

…in some local authorities where the sexual health service is no longer doing cervical screening [it] will have a small impact but not a huge impact, in other areas, it will have a big impact on coverage, we'll see activity go down around it, because the workload is

For peer review only - http://bmjopen.bmj.com/site/about/guidelines.xhtml

> just going to come straight back to primary care, and in different areas primary care didn't realise this was happening, the re-commissioning, hasn't got the capability and the capacity… [18352, PHE/NHSE, Area 1, Jan 2017]

Another screening consultant reflected that changes to NHSE 'footprints' (i.e. the abolition of Area Teams and the new, more regional focus of the organisation) had implications for the provision of cervical screening:

> …say we wanted to sort out cervical screening coverage in GP practices, in [name of PCT] you've got [*x*] GP practices, bottom 20 per cent you could talk to the [*y*] practices. In my new patch we've got [many more than *x*] practices. So you have to think in a completely different way. [17685, NHSE, Area 2, Dec 2016]

Several participants from different localities in both geographical areas pointed to long-standing challenges in ensuring good uptake rates for screening amongst their diverse local populations. There were concerns that these challenges would be exacerbated by a reduction in choice for women about where they could go for cervical screening tests:

> …you should have an integrated sexual health service where predominantly women can go in and get seen in one episode, in one place for all their sexual health needs, be that sexually transmitted infection testing and treatment and contraception. So I think probably in the past people worked very hard to get things like cervical screening into these services so that the needs of those women who perhaps wouldn't go to their local GP could be met in an environment they felt happy with. My feeling is now… that perhaps the type of women who traditionally would have gone for cervical screening [at their sexual health clinic] might not feel so comfortable in that environment [of the GP practice]. So particularly, say, a lady from a South Asian background who goes to a single handed male GP with no practice nurse, that's the kind of traditional person who might have gone to a family planning clinic for their cervical screening. [9742, local authority, Area 2, Jan 2016]

Summary

The HSCA separated commissioning responsibilities for some types of services, including sexual health. Our study participants told us that this had introduced complexity and confusion surrounding cervical screening commissioning, and they expressed concern that screening rates would decline as a result, with some areas potentially affected more than others due to differences in local contextual conditions. In order to explore this further, we designed a quantitative analysis to test the

For peer review only - http://bmjopen.bmj.com/site/about/guidelines.xhtml

proposition that CCGs most affected by this increase in complexity would have a greater decline in screening rates. Based upon the findings from our interviews that working with more than one local authority acted to increase the complexity associated with the commissioning role, we compared screening rates between those CCGs which relate to a single local authority and those required to work with two or more local authorities.

Quantitative findings

There were 14.1 million women eligible for screening in England in 2016 (NHS Digital 2016). Cervical screening rates decreased over time and the decline pre-dated the implementation of the HSCA in April 2013. Unassisted delivery rates also declined over time. The relative decline between the first year (2009/10) and the last year (2015/16) for unassisted deliveries (-4.17%) was larger than for cervical screening (-2.70%) (Table 3).

The changes in cervical screening rates over time were similar for practices in CCGs dealing with a single local authority (-2.53%) compared with practices in CCGs working with multiple local authorities (-2.87%). Figure 1 illustrates the trends in rates of cervical screening in the pre and post intervention periods for CCGs depending on the number of local authorities they worked with. There is a noticeable and sharp decline in the rates in both groups between 2011-12 and 2012-13.

For peer review only - http://bmjopen.bmj.com/site/about/guidelines.xhtml

Table 3: Numbers of general practices and mean rates of cervical screening and unassisted deliveries by year and by the number of local authorities (LAs) with which CCGs had to coordinate commissioning

Year	Number of general practices			Cervical screening rate (%)			Unassisted delivery rate (%)		
	1 LA	*2+ LAs*	*All*	*1 LA*	*2+ LAs*	*All*	*1 LA*	*2+ LAs*	*All*
2009/10	4,260	3,399	7,659	82.85	84.74	83.75	63.44	63.8	63.59
2010/11	4,261	3,403	7,664	82.74	84.61	83.63	63.19	63.76	63.44
2011/12	4,260	3,400	7,660	81.49	83.13	82.27	62.41	63.61	62.94
2012/13	4,249	3,399	7,648	81.28	82.87	82.04	62.17	63.08	62.57
2013/14	4,199	3,355	7,554	81.31	82.5	81.88	61.33	62.43	61.82
2014/15	4,125	3,302	7,427	81.21	82.54	81.85	60.67	61.98	61.25
2015/16	4,026	3,236	7,262	80.75	82.31	81.49	60.47	61.53	60.94
Relative change between 2009/10 and 2015/16 (%)				*-2.53*	*-2.87*	*-2.70*	*-4.68*	*-3.56*	*-4.17*

Notes: Mean cervical screening and unassisted delivery rates are weighted by the denominators used in the calculation of the rates. These are the eligible populations; the number of women aged between 25 and 64 years or the number of maternal deliveries.

For peer review only

Figure 1: Uptake (%) of cervical screening pre and post HSCA

[FIGURE 1 ABOUT HERE]

For peer review only

For peer review only - http://bmjopen.bmj.com/site/about/guidelines.xhtml

Comparing the unadjusted averages for the pre-HSCA and post-HSCA years, cervical screening rates decreased by 0.39% more for GP practices located in CCGs working with multiple local authorities compared to practices in CCGs working with a single local authority. Unassisted birth rates decreased by 0.40% less for GP practices in CCGs working with multiple local authorities compared to GP practices in CCGs working with a single local authority. As maternity services were largely unaffected by the HSCA, we assumed that these differential changes captured the unmeasured population influences that confound comparisons of the changes in the two groups of CCGs. Relative to the decreases in unassisted delivery rates, GP practices in CCGs working with multiple local authorities experienced a decrease in cervical screening rates of 0.79% compared to practices in CCGs working with a single local authority (Table 4).

The results were qualitatively similar when we estimated the formal triple-difference (for all years and 2011/12 onwards only) and lagged dependent variable regression models (Table 5). The triple-difference estimates show that there was a differentially larger decline of 0.62% [95% confidence interval: -0.941 to -0.297] (Model 1) in cervical screening rates for practices located in CCGs working with more than one local authority. The decrease is smaller using the shorter pre-period (0.259%; 95%CI -0.573 to 0.052, Model 2).

The direction of result is robust to the model specification and, although we rejected the assumption of parallel trends for Model 1 (all years), we could not reject the assumption for Model 2 (2011/12 onwards). We also found a similar result in Model 3 using the lagged dependent variable estimator, which yields unbiased estimates when pre-trends cannot be assumed to be parallel.

The results are also robust to different groupings of the number of local authorities that CCGs work with. Table 5 includes model estimates comparing CCGs working with one or two local authorities with CCGs working with more than two local authorities. The direction of results is equivalent; and the scale and significance are either equivalent or increased. The same pattern is repeated in terms of tests of parallel trends. We cannot reject the null hypothesis of parallel trends for Model 2 and the LDV estimation is preferable to Model 1 in which we can reject the null hypothesis of parallel trends.

For peer review only - http://bmjopen.bmj.com/site/about/guidelines.xhtml

Table 4: Rates of cervical screening and unassisted birth for CCGs working with one and more than one local authority (LA), before and after the introduction of the HSCA

**Condition**	**CCGs working with one LA**			**CCGs working with more than one LA**			**Difference between change in CCGs working with more than one LA and change in CCGs working with one LA**	**Difference in changes for cervical screening minus difference in changes for unassisted births**
	Average in the Pre-HSCA years	Average in the Post-HSCA years	*Change*	Average in the Pre-HSCA years	Average in the Post-HSCA years	*Change*		
	Percent							
**Affected (Cervical screening rates)**	82.09	81.09	*-1.00*	83.84	82.45	*-1.39*	**-0.39**	**-0.78**
**Unaffected (Unassisted birth rates)**	62.80	60.82	*-1.98*	63.56	61.98	*-1.58*	**0.40**	

Notes: values for pre and post are averages for all years in pre and post periods. The averages are weighted by the denominators used in the calculation of the rates. These are the eligible populations; the number of women aged between 25 and 64 years or the number of maternal deliveries.

For peer review only - http://bmjopen.bmj.com/site/about/guidelines.xhtml

Table 5: Triple-difference regression results†

	Difference-in-differences models				Lagged dependent variable models¤	
	All years (1)		2011/12 onwards (2)		All years (3)	
	Coefficient	*95% CI*	*Coefficient*	*95% CI*	*Coefficient*	*95% CI*
**Triple differences‡**	-0.617***	[-0.941; -0.297]	-0.259	[-0.573; 0.052]	-0.238**	[-0.446; -0.031]
**Triple differences‡ (Alternative grouping of CCGs®)**	-0.774***	[-1.134; -0.420]	-0.440**	[-0.786; -0.099]	-0.234**	[-0.461; -0.009]
**Number of observations**	105,745		75,099		44,472	
**Test of parallel trends**	**F (3, 7672)**	**p-value**	**F (1, 7667)**	**p-value**	**N/A**	
	4.89	0.002	0.33	0.567		

*Notes: ***indicates p < 0.01; **indicates p < 0.05; *indicates p < 0.1.*

†*Values are regression estimations from weighted least squares models on the empirical logit transformation of the rate including practice-condition specific fixed effects, full interaction of year with condition; and full interaction of year with the dummy for (N of LAs). Weighted by the denominators used to calculate the rates. Robust standard errors, clustered by practice.*

‡*The triple difference represents (the change over time in cervical screening rates for CCGs working with only one LA minus the change over time in cervical screening rates for CCGs working with more than one LA) minus (the change over time in unassisted birth rates for CCGs working with only one LA minus the change over time in unassisted birth rates for CCGs working with more than one LA).*

¤ *LDV also contains values of the dependent variable in each of the pre intervention years. Estimated only on post intervention years.*

® *This model uses an alternative grouping of CCGs based on the number of LAs they work with [(1 or 2) vs. (more than 2)]*

For peer review only - http://bmjopen.bmj.com/site/about/guidelines.xhtml

DISCUSSION

We conducted a mixed methods study exploring the impact of changes associated with the HSCA in the English NHS on cervical screening rates. We carried out qualitative interviews with senior figures from a variety of relevant organisations in two large, socioeconomically diverse areas of England. Analysis of these interviews suggested that cervical screening commissioning had become more complex, with responsibilities between organisations less certain, as a consequence of the HSCA. Some interviewees predicted there would be a reduction in cervical screening rates in particular areas. These findings prompted the development of an analysis to explore these issues quantitatively via a triple difference regression analysis of publically available data on cervical screening activity. To control for unmeasured confounders, we compared cervical screening rates with trends in unassisted birth rates because the commissioning of maternity services was unchanged pre- and post-HSCA.

Interviewees suggested a number of factors that might contribute to a reduction in cervical screening activity. Sexual health service commissioning responsibility had shifted to local authorities while NHS England was made responsible for commissioning national screening services, including cervical screening. Faced with financial austerity and cuts to their budgets, many local authorities were retendering their sexual health services with sexual health service providers but not including routine cervical screening. NHS England was also seemingly reluctant to commission sexual health clinics to perform cervical screening tests because this would entail a multitude of low-value contracts with numerous providers. This would be administratively laborious and practically difficult given the large size of NHSE's administrative areas and small numbers of NHSE commissioning staff in each area.

The quantitative analysis was designed to explore whether cervical screening activity had declined in areas most affected by commissioning organisational change. GP practices located in CCGs dealing with multiple local authorities, and therefore most exposed to increased commissioning complexity and potential disruption in services because of the lack of clarity of the roles of different organisations, experienced a larger decrease over time in cervical screening rates compared with practices in CCGs dealing with a single local authority. The opposite pattern was observed for unassisted births, which decreased more over time in the CCGs dealing with a single local authority. The triple-difference analyses confirmed that the effects were statistically significant and robust to different model specifications.

For peer review only - http://bmjopen.bmj.com/site/about/guidelines.xhtml

We have demonstrated unintended consequences arising out of a large-scale health system reform. Taken together, our findings suggest that there is an urgent need for clarification as to who holds the budget, and therefore who should be commissioning, cervical screening in the English NHS, and for local agreements to ensure that issues over funding and budgets do not disrupt screening programmes. More broadly, the issues we have identified in this study are of value to policy makers and system leaders in other health systems. The current study suggests that there are particular problems associated with service commissioning where co-ordination is required between multiple commissioners. This suggests that future commissioning reforms should include assessment of the likely impact on co-ordination, and a presumption in favour of commissioning all required services for geographical populations where possible. This may also have implications for mixed health systems, in which multiple payers (including public and private insurers as well as out of pocket payments) are responsible for services. In such systems achieving desirable population coverage for services such as screening may require specific co-ordination efforts.

Potential confounders and study strengths

We took 2009 as our starting point for pre-HSCA cervical screening activity. Two potential confounders to our results were considered. First, the high-profile case of Jade Goody, a 27-year-old reality TV star who was diagnosed with cervical cancer in August 2008 and died in March 2009. The contemporaneous media attention and publicity was linked with a substantial increase in cervical screening rates (around an extra half a million women) during the time between Goody's diagnosis and death. However, previous impacts of high-profile cases of celebrity cancer diagnoses upon population behaviour have tended to be brief and immediate rather than longer-lasting, and, therefore, we are confident that from 2010, rates of cervical screening returned towards underlying trends.[21] Second, the UK's Human Papillomavirus (HPV) vaccination programme was introduced in 2008 in order to reduce the incidence of cervical cancer.[22] The vaccine is offered to all girls aged 12-13 years, and figures for 2008-2014 show high uptake rates of just under 90%. It is likely that this vaccination programme will contribute to a reduction in cervical screening activity in future. However, the first cohort of women in the programme, i.e. those aged 12-13 in 2008, were only 21-22 years old in 2016/17, hence too young to have been invited for routine cervical screening (which begins at age 25) at the time of the study. We can, therefore, be confident that any changes to cervical screening rates cannot yet be attributed directly to the HPV programme, but any future research into cervical screening rates needs to take this into account.

We considered whether the results were sensitive to the group of CCGs in terms of the number of local authorities they worked with. The direction of results was the same, and the strength and

For peer review only - http://bmjopen.bmj.com/site/about/guidelines.xhtml

significance was increased, comparing CCGs working with one or two local authorities with those CCGs working with more than two local authorities. We also considered whether the results were sensitive to the choice of comparison indicator (unassisted births) for maternity services. We tested whether the results would hold for another indicator of maternity services: the rate of deliveries by caesarean section. We observed the same direction and significance of results for this indicator as well.

The average age of mothers at delivery is likely to be younger than the average age of women attending for cervical screening. For our analysis, we require that differential changes in maternity indicators between CCGs with simple and CCGs with complex local authority relationships are a good proxy for other factors influencing cervical screening rates. We have confirmed the empirical validity of this assumption by looking for parallel trends in the period before the HSCA, but we can never be entirely sure of its validity.

The findings presented here come from a longitudinal study of major healthcare system reform conducted by a multidisciplinary research team. The nature of this study facilitated the development of the relatively novel, sequential mixed methods approach in which the claims made in qualitative interviews could be tested in a subsequent quantitative analysis. There is a reinforcing effect in this analytical approach, which provides a strong cumulative indication that in areas of the country where complexity and coordination issues linked to the HSCA were more likely to occur there was an associated reduction in cervical screening rates.

ACKNOWLEDGMENTS

The authors would like to thank the research participants for their involvement, and acknowledge the valuable advice of the Project Advisory Group.

For peer review only - http://bmjopen.bmj.com/site/about/guidelines.xhtml

REFERENCES

[1] Best A, Greenhalgh T, Lewis S, Saul JE, Carroll S, Bitz J. Large-system transformation in health care: a realist review. Milbank Quarterly 2012;90:421–56. doi:10.1111/j.1468-0009.2012.00670.x.

[2] Edwards N. The triumph of hope over experience: Lessons from the history of reorganisation. NHS Confederation; 2010.

[3] Lale AS, Temple JMF. Has NHS reorganisation saved lives? A CuSum study using 65 years of data. J R Soc Med 2016;109:18–26. doi:10.1177/0141076815608853.

[4] Department of Health. The NHS Plan: a plan for investment, a plan for reform. London: The Stationery Office; 2000.

[5] Propper C. Market structure and prices: The responses of hospitals in the UK National Health Service to competition. Journal of Public Economics 1996;61:307–35. doi:10.1016/0047-2727(95)01561-2.

[6] Barbour R. The case for combining qualitative and quantitative approaches in health services research. J Health Serv Res Policy 1999;4:39–43. doi:10.1177/135581969900400110.

[7] Health and Social Care Act. (c.7). London: HMSO: 2012.

[8] Ham C, Baird B, Gregory S, Jabbal J, Alderwick H. The NHS under the coalition government. Part one: NHS reform. London: 2015.

[9] Peckham S, Falconer J, Gillam S, Hann A, Kendall S, Nanchahal K, et al. The organisation and delivery of health improvement in general practice and primary care: a scoping study. National Institute for Health Research; 2015.

[10] Weiss CH. Evaluation: methods for studying programs and policies. New Jersey: Prentice Hall; 1998.

[11] Department of Health. Equity and excellence: liberating the NHS. London: The Stationery Office; 2010.

[12] NHS England, Public Health England. Immunisation and screening national delivery framework & local operating model. 2013.

[13] Peckham S, Gadsby E, Jenkins L, Coleman A, Bramwell D, Perkins N. Views of public health leaders in English local authorities – changing perspectives following the transfer of responsibilities from the National Health Service to local government. Local Government Studies 2017;43:842–63. doi:10.1080/03003930.2017.1322069.

[14] Guest G. Describing mixed methods research: an alternative to typologies. Journal of Mixed Methods Research 2013;7:141–51. doi:10.1177/1558689812461179.

[15] Checkland K, Hammond J, Sutton M, Coleman A, Allen P, Mays N, et al. Understanding the new commissioning system in England: contexts, mechanisms and outcomes. Final report. PR-R6-1113-25001: NIHR Policy Research Programme; 2018.

[16] Braun V, Clarke V. Using thematic analysis in psychology. Qualitative Research in Psychology 2006;3:77–101. doi:10.1191/1478088706qp063oa.

[17] Imbens GW, Wooldridge JM. Recent developments in the econometrics of program evaluation. Journal of Economic Literature 2009;47:5–86. doi:10.1257/jel.47.1.5.

[18] O'Neill S, Kreif N, Grieve R, Sutton M, Sekhon JS. Estimating causal effects: considering three alternatives to difference-in-differences estimation. Health Serv Outcomes Res Methodol 2016;16:1–21. doi:10.1007/s10742-016-0146-8.

[19] Stevens S, Valderas JM, Doran T, Perera R, Kontopantelis E. Analysing indicators of performance, satisfaction, or safety using empirical logit transformation. BMJ 2016;352:i1114. doi:10.1136/bmj.i1114.

[20] Rogers W. sg17: Regression standard errors in clustered samples. Stata Technical Bulletin 1993;3:19–23.

For peer review only - http://bmjopen.bmj.com/site/about/guidelines.xhtml

[21] Lancucki L, Sasieni P, Patnick J, Day T, Vessey M. The impact of Jade Goody's diagnosis and death on the NHS Cervical Screening Programme. J Med Screen 2012;19:89–93. doi:10.1258/jms.2012.012028.
[22] Public Health England. Human Papillomavirus (HPV) Vaccine Coverage in England, 2008/09 to 2013/14. 2015.

FOOTNOTES

Author statement / Contributors: KC designed the study with input from MS, NM, PA, AC. JH, AH, LWG gathered and analysed the qualitative data. TM and MS designed the quantitative evaluation and conducted this analysis. JH drafted the manuscript to which all authors made substantial contributions. All authors approved the final version and agree to be accountable for all aspects of the analysis.

Funding: The report is based on independent research commissioned and funded by the NIHR Policy Research Programme ('Understanding the new commissioning system in England: contexts, mechanisms and outcomes', PR-R6-1113-25001). The views expressed in the publication are those of the author(s) and not necessarily those of the NHS, the NIHR, the Department of Health, arm's-length bodies or other government departments.

Competing interests: None declared.

Patient consent: Not required.

Ethics approval: Ethical approval was granted by one of The University of Manchester Research Ethics Committees (application 15085) in March 2015. Participants were provided written information about the study, provided written consent, or gave consent verbally at the beginning of telephone interviews.

Data sharing statement: No additional data are available.

For peer review only

For peer review only - http://bmjopen.bmj.com/site/about/guidelines.xhtml

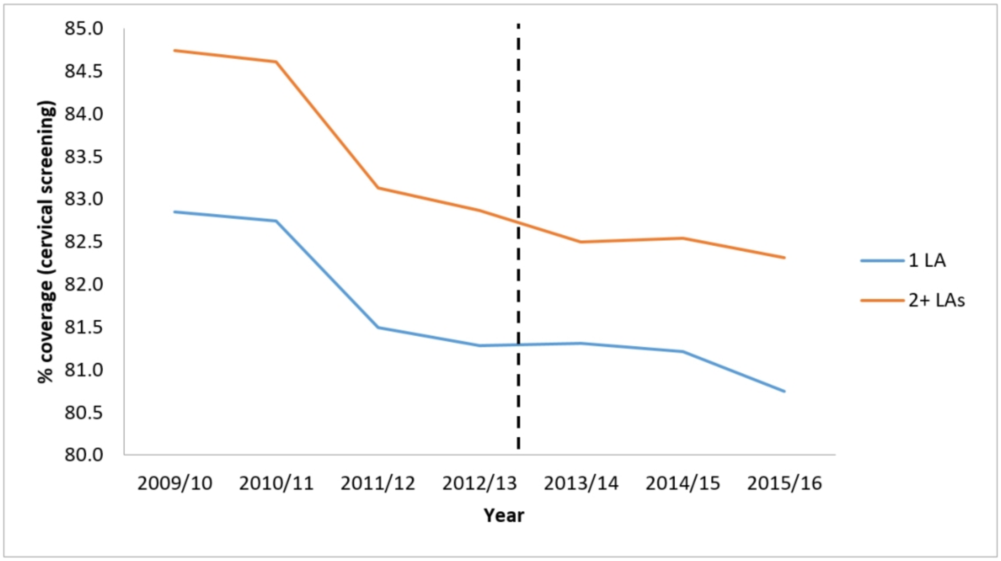

Figure 1: Uptake (%) of cervical screening pre and post HSCA

112x62mm (300 x 300 DPI)

Technical Appendix

Algebraic expressions for the estimated models

Triple-difference model

The triple-difference model can be written algebraically as:

$$y_{cjt} = \alpha + \phi L_j + \theta \pi_c + \rho L_j \pi_c + \sum_{t=2}^{T} \delta_t + \sum_{t=2}^{T} \psi_t \pi_c + \sum_{t=2}^{T} \gamma_t L_j + \beta L_j \pi_c D_t^* + \varepsilon_{cjt}$$

in which y_{cjt} is the value of indicator c in practice j and year t, L_j is a binary indicator for practices located in exposed areas, π_c is a dummy variable for the intervention indicator, $t_2 \dots t_T$ are dummy variables for years, and D_t^* is a dummy variable taking a value of one in post-implementation period. The coefficient of interest is β, the coefficient on the three-way interaction between the exposed area, the intervention indicator and the post-implementation period.

Lagged Dependent Variable model

The Lagged Dependent Variable model can be written algebraically as:

$$y_{cjt} = \alpha + \phi L_j + x \pi_c + \rho L_j \pi_c + \sum_{t=t^*}^{T} \delta_t + \sum_{t=t^*}^{T} \psi_t \pi_c + \sum_{k=1}^{t^*-1} \mu_k y_{cjk} + \sum_{k=1}^{t^*-1} \vartheta_k \pi_c y_{cjk} + \varepsilon_{cjt}$$

in which y_{cjt} is the value of indicator c in practice j and year t, L_j is a binary indicator for practices located in exposed areas, π_c is a binary indicator for the intervention indicator, and y_{cjk} are lagged values of the dependent variable in the pre-period ending at $t=t^*-1$. The model is estimated on data from the post period only and the ρ coefficient on the $L_j \pi_c$ term is the coefficient of interest.

For peer review only

BMJ Open

Exploring the impacts of the 2012 Health and Social Care Act reforms to commissioning upon clinical activity in the English NHS: A mixed methods study of cervical screening

Journal:	*BMJ Open*
Manuscript ID	bmjopen-2018-024156.R2
Article Type:	Research
Date Submitted by the Author:	08-Feb-2019
Complete List of Authors:	Hammond, Jonathan; University of Manchester, Division of Population Health, Health Services Research, and Primary Care; School of Health Sciences; Faculty of Biology, Medicine and Health<br>Mason, Thomas; University of Manchester, Manchester Centre for Health Economics; Division of Population Health, Health Services Research, and Primary Care; School of Health Sciences; Faculty of Biology, Medicine and Health<br>Sutton, Matt; University of Manchester, Division of Population Health, Health Services Research, and Primary Care; School of Health Sciences; Faculty of Biology, Medicine and Health<br>Hall, Alex; University of Manchester, Division of Nursing, Midwifery & Social Work; School of Health Sciences<br>Mays, Nicholas; London School of Hygiene and Tropical Medicine, Public Health and Policy<br>Coleman, Anna; University of Manchester, Division of Population Health, Health Services Research, and Primary Care; School of Health Sciences; Faculty of Biology, Medicine and Health<br>Allen, Pauline; Health Services Research Unit<br>Warwick-Giles, Lynsey; University of Manchester, Division of Population Health, Health Services Research, and Primary Care; School of Health Sciences; Faculty of Biology, Medicine and Health<br>Checkland, Kath; University of Manchester, Division of Population Health, Health Services Research, and Primary Care; School of Health Sciences; Faculty of Biology, Medicine and Health
**Primary Subject Heading**:	Health policy
Secondary Subject Heading:	Sexual health, Health services research, Public health, Health economics
Keywords:	NHS, Health policy < HEALTH SERVICES ADMINISTRATION & MANAGEMENT, Health system reform, Screening, Mixed methods, Commissioning

Exploring the impacts of the 2012 Health and Social Care Act reforms to commissioning upon clinical activity in the English NHS: A mixed methods study of cervical screening

Authors

- *Dr Jonathan Hammond [Corresponding author]*

Division of Population Health, Health Services Research, and Primary Care; School of Health Sciences; Faculty of Biology, Medicine and Health; Williamson Building (6th floor), The University of Manchester, Manchester, M13 9PL, UK
Email: jonathan.hammond@manchester.ac.uk; Tel.: 0161 2757648

- *Mr Thomas Mason*

Manchester Centre for Health Economics; Division of Population Health, Health Services Research, and Primary Care; School of Health Sciences; Faculty of Biology, Medicine and Health; The University of Manchester, Manchester, UK

- *Prof Matt Sutton*

Division of Population Health, Health Services Research, and Primary Care; School of Health Sciences; Faculty of Biology, Medicine and Health; The University of Manchester, Manchester, UK

- *Dr Alex Hall*

Division of Nursing, Midwifery and Social Work; School of Health Sciences; The University of Manchester, Manchester, UK

- *Prof Nicholas Mays*

Department of Health Services Research and Policy; Faculty of Public Health and Policy; London School of Hygiene and Tropical Medicine, London, UK

- *Dr Anna Coleman*

Division of Population Health, Health Services Research, and Primary Care; School of Health Sciences; Faculty of Biology, Medicine and Health; The University of Manchester, Manchester, UK

- *Prof Pauline Allen*

Department of Health Services Research and Policy; Faculty of Public Health and Policy; London School of Hygiene and Tropical Medicine, London, UK

- *Dr Lynsey Warwick-Giles*

Division of Population Health, Health Services Research, and Primary Care; School of Health Sciences; Faculty of Biology, Medicine and Health; The University of Manchester, Manchester, UK

- *Prof Kath Checkland*

Division of Population Health, Health Services Research, and Primary Care; School of Health Sciences; Faculty of Biology, Medicine and Health; The University of Manchester, Manchester, UK

For peer review only - http://bmjopen.bmj.com/site/about/guidelines.xhtml

ABSTRACT

Objectives: Explore the impact of changes to commissioning introduced in England by the Health and Social Care Act 2012 (HSCA) on cervical screening activity in areas identified empirically as particularly affected organisationally by the reforms.

Methods: Qualitative followed by quantitative methods. Qualitative: semi-structured interviews (with NHS commissioners, managers, clinicians, senior administrative staff from Clinical Commissioning Groups (CCGs), local authorities, service providers), observations of commissioning meetings in two metropolitan areas of England. Quantitative: triple-difference analysis of national administrative data. Variability in the expected effects of HSCA on commissioning was measured by comparing CCGs working with one local authority with CCGs working with multiple local authorities. To control for unmeasured confounders, differential changes over time in cervical screening rates (amongst women, 25-64 years) between CCGs more and less likely to have been affected by HSCA commissioning organisational change were compared with another outcome – unassisted birth rates – largely unaffected by HSCA changes.

Results: Interviewees identified that cervical screening commissioning and provision was more complex and 'fragmented', with responsibilities less certain, following the HSCA. Interviewees predicted this would reduce cervical screening rates in some areas more than others. Quantitative findings supported these predictions. Areas where CCGs dealt with multiple local authorities experienced a larger decline in cervical screening rates (1.4 percent) than those dealing with one local authority (1.0 percent). Over the same period, unassisted deliveries decreased by 1.6 percent and 2.0 percent, respectively, in the two groups.

Conclusions: Arrangements for commissioning and delivering cervical screening were disrupted and made more complex by the HSCA. Areas most affected saw a greater decline in screening rates than others. The fact that this was identified qualitatively and then confirmed quantitatively using strengthens this finding. The study suggests large scale health system reforms may have unintended consequences, and that complex commissioning arrangements may be problematic.

Keywords:

Screening; Health policy; Mixed methods; Commissioning; NHS; Health system reform

For peer review only - http://bmjopen.bmj.com/site/about/guidelines.xhtml

ARTICLE SUMMARY

Strengths and limitations of this study:

- Few studies have investigated in detail the impacts of large-scale health system change.
- This study combines detailed qualitative data exploring impacts on the system with quantitative exploration of important outcomes, supporting causal inference.
- Based on qualitative findings, we developed a quantitative measure for assessing the extent of disruption to the English NHS commissioning system as a result of the 2012 Health and Social Care Act.
- We found that cervical screening rates decreased more post-Act in areas that had experienced higher levels of disruption.

INTRODUCTION

Structural re-organisations of publicly financed health care systems, driven by central government or other state agencies, are frequently employed with the objective of improving health care delivery, and thus population health outcomes, whilst reducing or containing costs.[1] However, such endeavours can be disruptive and expensive.[2] It is important to understand what possible impacts these reorganisations have in order to understand their value.[3]

In the English NHS, attempts to evaluate the impact of reorganisations have typically used operational indicators (e.g. bed availability, numbers of staff) and measures of clinical activity because their improvement was the stated goal of government policy (e.g. The NHS Plan,[4]). Other studies have attempted to assess the impacts of reforms by measuring their effects on prices, quality and quantity of provision.[5] Most studies have relied upon quantitative analysis of measures that were explicitly targeted by policy reforms. There is a need for approaches which combine qualitative and quantitative methods to generate a deeper understanding of the impacts of structural reorganisation.[6]

The most recent structural reorganisation of the English NHS, the Health and Social Care Act[7] (hereafter 'HSCA' or 'the Act'), was introduced in April 2013 and included wide-ranging changes to the health services commissioning system. We explore whether changes to the commissioning of cervical screening services resulting from the Act affected uptake. This analysis uses a relatively novel mixed methods approach. An initial 'bottom-up' qualitative analysis allowed us to identify problematic issues associated with the HSCA for those working locally in the health service

For peer review only - http://bmjopen.bmj.com/site/about/guidelines.xhtml

commissioning system. This process highlighted the disruption to established commissioning arrangements and cervical screening as a clinical activity which may be specifically affected by this disruption. We then developed a quantitative investigation to explore this more fully. Together these analyses allow us to infer causation.

The HSCA and changes to cervical screening commissioning

The HSCA is regarded as one of the most wide-ranging legislative reforms in the history of the English NHS.[8] Primary Care Trusts (PCTs), 152 organisations previously responsible for the commissioning of primary, community and secondary health services from providers on behalf of local populations, were abolished. Their commissioning functions were split between three groups of organisations: 211 (now 195) newly created Clinical Commissioning Groups (CCGs), membership organisations constituted by GP (family doctor) practices, given responsibility for commissioning services for their local populations; NHS England, a new arm's-length governmental body with responsibility for authorising and assessing CCGs, and commissioning some services at a national level; and top-tier and single-tier elected local authorities, which took responsibility for the majority of public health services for the first time since 1974. In addition, Public Health England (PHE) was created as an executive agency of the Department of Health, to unify the diverse public health profession and provide expert support for local public health services.

In some service areas, the transfer of commissioning responsibilities was relatively straightforward (e.g. the commissioning of routine orthopaedic surgery was passed from PCTs to CCGs with minimal alteration to the bundle of services involved). In other service areas, the transfers were much more complex, particularly the commissioning of national screening programmes and sexual health services, as a result of changes to public health commissioning. Pre-HSCA, national screening programmes and sexual health services were both commissioned by PCTs. Cervical screening was largely provided by GP practices, which received additional funding linked to levels of activity,[9] but some women opted to have their cervical smears in PCT-commissioned sexual health clinics. Post-HSCA, responsibility for public health services, including most sexual health services, was transferred to local authorities. The underlying programme theory (i.e. the explicit expectation about how the policy would work;[10]) was that local authorities would be better placed to address the wider determinants of health and wellbeing than the NHS because they could link public health services with their existing responsibilities, such as for transport and housing.[11] NHS England took responsibility for commissioning national screening programmes.[8] NHS England's regional teams are responsible for commissioning screening programmes, supported by PHE staff 'embedded' within NHSE's screening and immunisation teams.[12] There was no identifiable underlying

For peer review only - http://bmjopen.bmj.com/site/about/guidelines.xhtml

programme theory for this specific change to screening programme commissioning. However, it is notable that, in contrast to the emphasis placed upon localism associated with the creation of CCGs and with the transfer of public health to local authorities, screening commissioning became more centralised as a consequence of the HSCA.

Table 1 shows the organisations with responsibilities of relevance to the commissioning of sexual health, including cervical cancer screening services, pre and post-HSCA. It illustrates how responsibility for such services, previously commissioned by PCTs, was split between different agencies. This increased complexity and fragmentation of responsibilities had the potential to disrupt service commissioning.[13]

For peer review only

For peer review only - http://bmjopen.bmj.com/site/about/guidelines.xhtml

Table 1: Organisations of significance to the commissioning of cervical screening pre and post HSCA

Pre-HSCA	
Primary Care Trusts	Responsible for all public health commissioning, including sexual health services; Responsible for commissioning national screening programmes, including cervical screening
UK National Screening Committee	Provision of advice and support to NHS organisations on population screening programmes
Post-HSCA	
Local authorities	Responsible for most public health commissioning, including most sexual health services (contraception over and above GP contract, testing and treatment of sexually transmitted infections, sexual health advice and promotion)
NHS England (NHSE)	Responsible for commissioning national screening programmes, including cervical screening, and some sexual health services (notably contraception through GP contract, and HIV treatment)
Public Health England (PHE) (including National Screening Committee)	Hosts UK National Screening Committee secretariat, which retains the same role; PHE staff embedded in sub-regional NHSE screening and immunisation teams to provide expertise and 'leadership'
Clinical Commissioning Groups (CCGs)	Responsible for improving clinical outcomes for their patients and prevent premature death (as part of the NHS Outcomes Framework, which CCGs are held to account on by NHSE); GP members are funded to carry out cervical screening through the GP Contract

This analysis comes from a study designed to foster emergent interplay between qualitative and quantitative data analysis.[14,15] The focus on cervical screening was not established at the project design stage but driven by the initial qualitative interview findings related to sexual health commissioning arrangements and screening activity post-HSCA. These findings prompted us to consider a quantitative exploration of predictions made by interviewees relating to potential changes in cervical screening activity.

METHODS

Study context and design

This analysis forms part of a longitudinal project, with data collected between January 2015 and December 2017, into the effect of the HSCA upon the commissioning system in England. We

For peer review only - http://bmjopen.bmj.com/site/about/guidelines.xhtml

combined a qualitative and quantitative exploration of the commissioning of services in two large, socioeconomically diverse metropolitan areas of England with a national level quantitative study of commissioning outcomes. We used a sequential mixed methods approach in which initial qualitative data collection and analysis were used to shape an ensuing quantitative analysis using routinely available data. We therefore present the qualitative and quantitative methods and findings in the order undertaken, and integrate them in the Discussion.

Patient and public involvement

Our interest in exploring the impact of systemic commissioning change on cervical screening rates was driven initially by concerns expressed by interviewees about potentially negative consequences for patients relating to new arrangements. Patients were not directly involved in the design of, or recruitment to, the overarching project, but an advisory group including a patient representative met regularly throughout the project and played an important role in supporting its development. We presented our initial qualitative findings relating to cervical screening, and early ideas for developing a mixed-methods investigation, to our advisory group and were encouraged by our patient representative to pursue this. The results of the broader project were disseminated to participants, and the advisory group, in the form of a series of short reports focusing on specific areas of commissioning and the final report.

Qualitative component

Setting, participants, sampling and data collection

The qualitative component took place between March 2015 and August 2017, focussing on two metropolitan 'health economies' covering a geographical population and a group of commissioning organisations and providers with close operational links. Across both areas, we conducted 143 interviews (each typically an hour in length), 93 of which related to sexual health commissioning, with clinical and non-clinical commisioners, managers, clinicians, and senior administrative staff from CCGs, local authorities, service providers and third sector organisations. Organisations and participants were sampled purposively for variation in type and role. We identified participants through organisational websites, personal contacts and through 'snowballing' in which we asked participants to recommend other potential participants. Additionaly, eight hours of meetings of an inter-organisaional sexual health co-ordinating group involving sexual health commissioners and providers were observed in one of the areas.

Interviews focused upon the commissioning system pre- and post-HSCA, exploring continuities and changes to personal and organisational roles, key issues and challenges, accountability and

For peer review only - http://bmjopen.bmj.com/site/about/guidelines.xhtml

performance management, interorganisational relationships and communication, and commissioning decision-making. Interviews took place either in person (usually in participants' offices) or over the telephone. All interviews were audio-recorded and all interviewees were provided with written information about the study before consenting to participate.

Data analysis

Audio recordings of interviews were transribed verbatim, and observational notes from meetings were produced contemporaneously. Transcripts and observational notes were imported into NVivo 10 software and analysed thematicly by JH and AH.[16] This involved repeated readings of transcripts to become sufficiently familiar with their contents, identifying initial codes and coding chunks of data, searching for themes and then iteratively defining and re-constituting themes. Our findings (see below) contained some predictions made by participants regarding changes in cervical screening activity as a consequence of the Act. This prompted us to explore these predictions in a quantitative analysis, which we now describe.

Quantitative component

Interviewees identified that the HSCA had introduced confusion over responsibility for the commissioning of cervical screening services, and had increased variability of provision. They hypothesised that cervical screening rates might be reduced by the new commissioning arrangements; this prompted discussions amongst the research team and advisory group about developing a way of testing these predictions quantitatively as far as routine data would permit. As the Act had been implemented in all areas simultaneously removing the scope for a quasi-experimental approach, we sought to identify a measure of variability in the extent to which the Act would have been expected to make commissioning more difficult in each area. One of the features of the post-HSCA system was that some, but not all, CCGs were established which crossed local authority boundaries. Some CCGs related to as many as three separate local authorities. Local authorities were now directly involved in sexual health services' commissioning. Findings revealed that CCGs experienced extra challenges when they had to engage with more than one local authority. This suggested that the burden of additional inter-organisational coordination might have consequences for commissioning.

As each local authority developed its own approach to cervical screening in its local sexual health clinics, we explored the possibility that GP practices located in CCGs which had to work with more than one local authority might experience lower screening rates compared with practices located in CCGs which had only to deal with one local authority. We compare the demographic characteristics

For peer review only - http://bmjopen.bmj.com/site/about/guidelines.xhtml

of these two groups in Table 2. The 89 CCGs dealing with more than one local authority had a slightly older population profile than the 119 CCGs which dealt with only one local authority but were otherwise highly comparable.

Table 2: CCG demographic characteristics depending on the number of local authorities that the CCG needs to work with

	One local authority	More than one local authority
Number of CCGs	119	89
Population (millions)	29.5	26.9
Female	50.0%	50.5%
Aged 0-9	12.3%	11.4%
Aged 10-19	11.2%	11.2%
Aged 20-39	30.0%	24.8%
Aged 40-59	26.5%	27.7%
Aged 60-79	16.0%	19.7%
Aged 80 and over	4.1%	5.3%

For peer review only

Because cervical screening rates may be influenced by other factors that we cannot observe and may change over time in different ways between the two groups of CCGs, we also compared screening rates with an outcome that was unlikely to have been affected by the introduction of HSCA. We used unassisted births (i.e. uncomplicated deliveries which did not require any intervention) as a percentage of all maternal deliveries, since the commissioning of maternity services was largely unchanged by the Act.

We applied a triple-difference approach. The triple difference represents (the change over time in cervical screening rates for CCGs working with only one local authority minus the change over time in cervical screening rates for CCGs working with more than one local authority) minus (the change over time in unassisted birth rates for CCGs working with only one local authority minus the change over time in unassisted birth rates for CCGs working with more than one local authority).

The screening rate is defined as the percentage of women aged between 25 and 64 years who had received a cervical screening test in the preceding five years. This indicator was derived from annual,

For peer review only - http://bmjopen.bmj.com/site/about/guidelines.xhtml

practice-level data from the Quality and Outcomes Framework (QOF), 2009-10 to 2015-16. The comparison indicator is unassisted births as a percentage of all maternal deliveries. This indicator was produced using operation codes in Hospital Episode Statistics (HES) for 2009-10 to 2015-16. We aggregated the spell-level data by general practice and financial year.

The key assumption underpinning the triple-difference estimator is that, conditional on the other variables in the model, the differences in the changes over time in the intervention indicator (cervical screening) between the 'exposed' and the 'control' areas (in this case, CCGs working with one local authority versus CCGs working with more than one) would have been the same as the differences in the changes over time in the comparison indicator (unassisted births) between the exposed and control areas in the absence of the intervention. This is a more complex version of the 'parallel trends' assumption required for the double-difference, or difference-in-differences, estimator.[17]

A popular test of this assumption in the double-difference case is that there are parallel trends over time in the outcomes in the intervention and comparison group in the pre-intervention period. For our triple-difference case, we used an F-test to assess the joint significance of interactions between the year effects and the binary variable representing the combination of exposed area and treated indicator in the pre-period.

We also used the lagged dependent variable (LDV) estimator. This model is estimated only on data in the post-intervention period and is a less biased estimator of treatment effects when the assumption of parallel pre-trends does not hold.[18] We set up the LDV model to generate the equivalent impact estimate as the triple-difference model. The model included: dummy variables for year; values of the dependent variable in each of the pre-intervention periods; a dummy variable classifying practices depending on whether they were located in CCGs working with more than one local authority; interactions between year and condition dummies; interactions between values of the dependent variable in the pre-intervention period and the condition dummy; and an interaction between the dummy variable classifying practices depending on whether they were located in CCGs working with more than one local authority and the condition dummy. The final term is the impact estimate, showing whether cervical screening was differentially affected after the introduction of the reforms for local authorities working with multiple CCGs.

We estimated the regression models in Stata 14.1 using dummy variable weighted least squares regression with fixed effects for practice-indicator combinations. The ways in which these models are estimated using regression analyses are described formally in the technical appendix. As the

For peer review only - http://bmjopen.bmj.com/site/about/guidelines.xhtml

dependent variable is a proportion, and constrained to lie between 0 and 1, we used the empirical logit transformation and back-transformed the coefficients and associated 95% confidence intervals using the mean value of the cervical screening rate.[19] We clustered the standard errors at the GP practice level.[20] The general form of the STATA command is: *areg {depvar} {indepvars} [aw=denom], robust absorb(practicexindicator) cluster(practice).*

RESULTS

Qualitative findings

Interviewees told us that CCGs working with more than one local authority experienced a number of challenges, including: finding sufficient capacity to engage in multiple meetings of the same type with different local authorities; managing additional collaborative relationships; working with organisations experiencing different financial pressures from each other with different approaches to public health spending; and attempting to develop integrated health and social care arrangements with one local authority that did not have unintended and undesirable consequences for plans with another. The following extract illustrates issues relating to difficulties commissioning a single service offer for CCG patients and the additional resources required for a CCG working with multiple local authorities. (Interview data extracts are denoted by square brackets with numerical participant ID, participant's organisation type, Area (1 or 2), and month and year of the interview.)

> We do have two sets of safeguarding arrangements. So I guess at one level, one can say there is a risk of and there are examples of services being subtly different. Equally, you've got to service two times the number of these processes, which can be quite labour-intensive. [2778, CCG, Area 1, April 2015]

In our analysis relating directly to issues surrounding the commissioning and provision of cervical screening post-HSCA, we identified two main themes: confusion and uncertainty regarding budgets and responsibilities, and potential impacts on cervical screening rates. Many of the issues discussed below are likely to be exacerbated when the number of interacting commissioning organisations in a local area are increased.

Confusion and uncertainty regarding budgets and responsibilities

Before the HSCA, both cervical screening and sexual health services were commissioned by PCTs. As one screening and immunisation lead outlined, cervical screening tests (sometimes referred to as smear tests) were provided by GP practices, but patients could usually also have them at sexual health clinics [17685, NHSE, Area 2, Dec 2016]. Whereas pre-HSCA PCTs held the budget for both

For peer review only - http://bmjopen.bmj.com/site/about/guidelines.xhtml

cervical screening and sexual health services, following the Act these budgets were separated. This meant that the local authority budget and responsibility for sexual health did not extend to cervical screening. One local authority public health consultant reported that, in spite of this, PHE was sending letters to patients explicitly stating that they could choose to attend either their GP practice or their local sexual health clinic for their cervical screening test. This highlights confusion regarding commissioning arrangements and budgetary responsibility:

> Public Health England were writing around to people saying …you're due your smear, you can go to your general practice or you can go to your local sexual health clinic. And we said, but we don't have the money for them to do that, they can't come here routinely unless you're going to pay us for that. Public Health England, the screening people, they have the money to pay for the smears. But in all the moving around of the budgets, the money for smears that were taken outside general practice doesn't seem to be anywhere [8384, local authority, Area 1, Nov 2015].

One participant from NHS England offered a different perspective. He argued that the public health budget of each local authority reflected the levels of cervical screening activity that had taken place in its sexual health clinics pre-HSCA. However, this is not clear because, in the past, the funding was not “disaggregated” [4058, NHSE, Area 1, Jun 2015]. Therefore, it is not possible to establish what the pre-HSCA sexual health component of the public health budget covered.

> …they [local authorities] think they’re not being paid for it [cervical screening]. But, actually, in truth, whatever they were doing at the point of transition if they were doing loads of cervical smears they were just doing loads of cervical smears, so they had the money. There wasn’t a problem when they were doing them before, it’s just the money wasn’t disaggregated. However local authorities have been put under significant pressure in their public health teams to reduce their budgets. So these kinds of things are examples where you can say it’s not our responsibility so therefore we’re taking that element out [4058, NHSE, Area 1, Jun 2015].

The above quote illustrates a phenomenon reported by a number of participants that local authorities had re-procured their sexual health services and had taken a position that they would not commission their sexual health provider(s) to do routine cervical screening, because it was not their commissioning responsibility. However, as one member of a screening team in Area 2 illustrated, NHSE was also reluctant to explicitly commission sexual

For peer review only - http://bmjopen.bmj.com/site/about/guidelines.xhtml

health services to provide cervical screening, seemingly because of administrative challenges relating to numerous low-value contracts with providers:

> So cervical screening, we could go to every sexual health provider and have a separate contract. The difficulty again becomes around commissioning capacity. So, I think we've got [x] local authorities, so we have [x] separate contracts all very low value, it's about 1,000 screens in each, so you're talking maybe [x] £20,000 contracts or something. So, it's a very bitty way of doing stuff. So, we could still do it and we could pay for it, but in terms of the amount of paperwork or the amount of outcomes it becomes potentially unmanageable. [17685, PHE/NHSE, Area 2, Dec 2016]

This participant went on to indicate that he would prefer local authorities to commission cervical screening as part of their sexual health contracts, but acknowledged the political difficulties for local authorities to justify spending money on an area of service that was not formally their responsibility, especially given the context of diminishing local authority budgets:

> In a way, wouldn't it be so much easier if the local authorities just included it as part of their normal service? But their argument would be that's not our role, and how can we defend to the [elected] councillors that we're spending money on stuff that we don't have to, that someone else is meant to be spending money on? And our argument is well, it's just so much simpler and it's not a lot of money. That's the kind of discussion. And it eventually ends up with them withdrawing money and us saying well, we're not buying it either then. [17685, PHE/NHSE, Area 2, Dec 2016]

Potential impacts on cervical screening rates

One local authority commissioner suggested that the policy of his local authority was to continue to facilitate opportunistic cervical screening tests at sexual health clinics, but not routine tests, because to provide the latter would have a detrimental impact upon other sexual health services that the local authority was now obligated to commission ("if we don't say no to [routine] smears, we'll be turning [other] people away, symptomatic patients away, or women needing contraception away. And that's our duty" [8384, local authority, Area 1, Nov 2015]). He reported that local CCGs complained about this discontinuation of routine cervical screening at sexual health clinics, because there was insufficient capacity within general practice for CCGs to meet their cervical screening targets, and thus they required sexual health clinics to provide a proportion of cervical screening

For peer review only - http://bmjopen.bmj.com/site/about/guidelines.xhtml

activity. One screening consultant developed this point by suggesting that some localities would see a substantial reduction in screening activity because of a lack of capacity within primary care:

> …in some local authorities where the sexual health service is no longer doing cervical screening [it] will have a small impact but not a huge impact, in other areas, it will have a big impact on coverage, we'll see activity go down around it, because the workload is just going to come straight back to primary care, and in different areas primary care didn't realise this was happening, the re-commissioning, hasn't got the capability and the capacity… [18352, PHE/NHSE, Area 1, Jan 2017]

Another screening consultant reflected that changes to NHSE 'footprints' (i.e. the abolition of Area Teams and the new, more regional focus of the organisation) had implications for the provision of cervical screening:

> …say we wanted to sort out cervical screening coverage in GP practices, in [name of PCT] you've got [*x*] GP practices, bottom 20 per cent you could talk to the [*y*] practices. In my new patch we've got [many more than *x*] practices. So you have to think in a completely different way. [17685, NHSE, Area 2, Dec 2016]

Several participants from different localities in both geographical areas pointed to long-standing challenges in ensuring good uptake rates for screening amongst their diverse local populations. There were concerns that these challenges would be exacerbated by a reduction in choice for women about where they could go for cervical screening tests:

> …you should have an integrated sexual health service where predominantly women can go in and get seen in one episode, in one place for all their sexual health needs, be that sexually transmitted infection testing and treatment and contraception. So I think probably in the past people worked very hard to get things like cervical screening into these services so that the needs of those women who perhaps wouldn't go to their local GP could be met in an environment they felt happy with. My feeling is now… that perhaps the type of women who traditionally would have gone for cervical screening [at their sexual health clinic] might not feel so comfortable in that environment [of the GP practice]. So particularly, say, a lady from a South Asian background who goes to a single handed male GP with no practice nurse, that's the kind of traditional person who might have gone to a family planning clinic for their cervical screening. [9742, local authority, Area 2, Jan 2016]

For peer review only - http://bmjopen.bmj.com/site/about/guidelines.xhtml

Summary

The HSCA separated commissioning responsibilities for some types of services, including sexual health. Our study participants told us that this had introduced complexity and confusion surrounding cervical screening commissioning, and they expressed concern that screening rates would decline as a result, with some areas potentially affected more than others due to differences in local contextual conditions. In order to explore this further, we designed a quantitative analysis to test the proposition that CCGs most affected by this increase in complexity would have a greater decline in screening rates. Based upon the findings from our interviews that working with more than one local authority acted to increase the complexity associated with the commissioning role, we compared screening rates between those CCGs which relate to a single local authority and those required to work with two or more local authorities.

Quantitative findings

There were 14.1 million women eligible for screening in England in 2016 (NHS Digital 2016). Cervical screening rates decreased over time and the decline pre-dated the implementation of the HSCA in April 2013. Unassisted delivery rates also declined over time. The relative decline between the first year (2009/10) and the last year (2015/16) for unassisted deliveries (-4.17%) was larger than for cervical screening (-2.70%) (Table 3).

The changes in cervical screening rates over time were similar for practices in CCGs dealing with a single local authority (-2.53%) compared with practices in CCGs working with multiple local authorities (-2.87%). Figure 1 illustrates the trends in rates of cervical screening in the pre and post intervention periods for CCGs depending on the number of local authorities they worked with. There is a noticeable and sharp decline in the rates in both groups between 2011-12 and 2012-13.

For peer review only

Table 3: Numbers of general practices and mean rates of cervical screening and unassisted deliveries by year and by the number of local authorities (LAs) with which CCGs had to coordinate commissioning

Year	Number of general practices			Cervical screening rate (%)			Unassisted delivery rate (%)		
	1 LA	*2+ LAs*	*All*	*1 LA*	*2+ LAs*	*All*	*1 LA*	*2+ LAs*	*All*
2009/10	4,260	3,399	7,659	82.85	84.74	83.75	63.44	63.8	63.59
2010/11	4,261	3,403	7,664	82.74	84.61	83.63	63.19	63.76	63.44
2011/12	4,260	3,400	7,660	81.49	83.13	82.27	62.41	63.61	62.94
2012/13	4,249	3,399	7,648	81.28	82.87	82.04	62.17	63.08	62.57
2013/14	4,199	3,355	7,554	81.31	82.5	81.88	61.33	62.43	61.82
2014/15	4,125	3,302	7,427	81.21	82.54	81.85	60.67	61.98	61.25
2015/16	4,026	3,236	7,262	80.75	82.31	81.49	60.47	61.53	60.94
Relative change between 2009/10 and 2015/16 (%)				*-2.53*	*-2.87*	*-2.70*	*-4.68*	*-3.56*	*-4.17*

Notes: Mean cervical screening and unassisted delivery rates are weighted by the denominators used in the calculation of the rates. These are the eligible populations; the number of women aged between 25 and 64 years or the number of maternal deliveries.

For peer review only - http://bmjopen.bmj.com/site/about/guidelines.xhtml

Figure 1: Uptake (%) of cervical screening pre and post HSCA

[FIGURE 1 ABOUT HERE]

For peer review only

For peer review only - http://bmjopen.bmj.com/site/about/guidelines.xhtml

Comparing the unadjusted averages for the pre-HSCA and post-HSCA years, cervical screening rates decreased by 0.39% more for GP practices located in CCGs working with multiple local authorities compared to practices in CCGs working with a single local authority. Unassisted birth rates decreased by 0.40% less for GP practices in CCGs working with multiple local authorities compared to GP practices in CCGs working with a single local authority. As maternity services were largely unaffected by the HSCA, we assumed that these differential changes captured the unmeasured population influences that confound comparisons of the changes in the two groups of CCGs. Relative to the decreases in unassisted delivery rates, GP practices in CCGs working with multiple local authorities experienced a decrease in cervical screening rates of 0.79% compared to practices in CCGs working with a single local authority (Table 4).

The results were qualitatively similar when we estimated the formal triple-difference (for all years and 2011/12 onwards only) and lagged dependent variable regression models (Table 5). The triple-difference estimates show that there was a differentially larger decline of 0.62% [95% confidence interval: -0.941 to -0.297] (Model 1) in cervical screening rates for practices located in CCGs working with more than one local authority. The decrease is smaller using the shorter pre-period (0.259%; 95%CI -0.573 to 0.052, Model 2).

The direction of result is robust to the model specification and, although we rejected the assumption of parallel trends for Model 1 (all years), we could not reject the assumption for Model 2 (2011/12 onwards). We also found a similar result in Model 3 using the lagged dependent variable estimator, which yields unbiased estimates when pre-trends cannot be assumed to be parallel.

The results are also robust to different groupings of the number of local authorities that CCGs work with. Table 5 includes model estimates comparing CCGs working with one or two local authorities with CCGs working with more than two local authorities. The direction of results is equivalent; and the scale and significance are either equivalent or increased. The same pattern is repeated in terms of tests of parallel trends. We cannot reject the null hypothesis of parallel trends for Model 2 and the LDV estimation is preferable to Model 1 in which we can reject the null hypothesis of parallel trends.

For peer review only - http://bmjopen.bmj.com/site/about/guidelines.xhtml

Table 4: Rates of cervical screening and unassisted birth for CCGs working with one and more than one local authority (LA), before and after the introduction of the HSCA

Condition	CCGs working with one LA			CCGs working with more than one LA			Difference between change in CCGs working with more than one LA and change in CCGs working with one LA	Difference in changes for cervical screening minus difference in changes for unassisted births
	Average in the Pre-HSCA years	Average in the Post-HSCA years	*Change*	Average in the Pre-HSCA years	Average in the Post-HSCA years	*Change*		
	Percent							
**Affected (Cervical screening rates)**	82.09	81.09	*-1.00*	83.84	82.45	*-1.39*	**-0.39**	**-0.78**
**Unaffected (Unassisted birth rates)**	62.80	60.82	*-1.98*	63.56	61.98	*-1.58*	**0.40**	

Notes: values for pre and post are averages for all years in pre and post periods. The averages are weighted by the denominators used in the calculation of the rates. These are the eligible populations; the number of women aged between 25 and 64 years or the number of maternal deliveries.

For peer review only - http://bmjopen.bmj.com/site/about/guidelines.xhtml

Table 5: Triple-difference regression results†

	Difference-in-differences models				Lagged dependent variable models¤	
	**All years (1)**		**2011/12 onwards (2)**		**All years (3)**	
	Coefficient	*95% CI*	*Coefficient*	*95% CI*	*Coefficient*	*95% CI*
**Triple differences‡**	-0.617***	[-0.941; -0.297]	-0.259	[-0.573; 0.052]	-0.238**	[-0.446; -0.031]
**Triple differences‡ (Alternative grouping of CCGs®)**	-0.774***	[-1.134; -0.420]	-0.440**	[-0.786; -0.099]	-0.234**	[-0.461; -0.009]
**Number of observations**	105,745		75,099		44,472	
**Test of parallel trends**	**F (3, 7672)**	**p-value**	**F (1, 7667)**	**p-value**	**N/A**	
	4.89	0.002	0.33	0.567		

*Notes: ***indicates p < 0.01; **indicates p < 0.05; *indicates p < 0.1.*

†*Values are regression estimations from weighted least squares models on the empirical logit transformation of the rate including practice-condition specific fixed effects, full interaction of year with condition; and full interaction of year with the dummy for (N of LAs). Weighted by the denominators used to calculate the rates. Robust standard errors, clustered by practice.*

‡*The triple difference represents (the change over time in cervical screening rates for CCGs working with only one LA minus the change over time in cervical screening rates for CCGs working with more than one LA) minus (the change over time in unassisted birth rates for CCGs working with only one LA minus the change over time in unassisted birth rates for CCGs working with more than one LA).*

¤ *LDV also contains values of the dependent variable in each of the pre intervention years. Estimated only on post intervention years.*

® *This model uses an alternative grouping of CCGs based on the number of LAs they work with [(1 or 2) vs. (more than 2)]*

For peer review only - http://bmjopen.bmj.com/site/about/guidelines.xhtml

For peer review only

DISCUSSION

We conducted a mixed methods study exploring the impact of changes associated with the HSCA in the English NHS on cervical screening rates. We carried out qualitative interviews with senior figures from a variety of relevant organisations in two large, socioeconomically diverse areas of England. Analysis of these interviews suggested that cervical screening commissioning had become more complex, with responsibilities between organisations less certain, as a consequence of the HSCA. Some interviewees predicted there would be a reduction in cervical screening rates in particular areas. These findings prompted the development of an analysis to explore these issues quantitatively via a triple difference regression analysis of publically available data on cervical screening activity. To control for unmeasured confounders, we compared cervical screening rates with trends in unassisted birth rates because the commissioning of maternity services was unchanged pre- and post-HSCA.

Interviewees suggested a number of factors that might contribute to a reduction in cervical screening activity. Sexual health service commissioning responsibility had shifted to local authorities while NHS England was made responsible for commissioning national screening services, including cervical screening. Faced with financial austerity and cuts to their budgets, many local authorities were retendering their sexual health services with sexual health service providers but not including routine cervical screening. NHS England was also seemingly reluctant to commission sexual health clinics to perform cervical screening tests because this would entail a multitude of low-value contracts with numerous providers. This would be administratively laborious and practically difficult given the large size of NHSE’s administrative areas and small numbers of NHSE commissioning staff in each area.

The quantitative analysis was designed to explore whether cervical screening activity had declined in areas most affected by commissioning organisational change. GP practices located in CCGs dealing with multiple local authorities, and therefore most exposed to increased commissioning complexity and potential disruption in services because of the lack of clarity of the roles of different organisations, experienced a larger decrease over time in cervical screening rates compared with practices in CCGs dealing with a single local authority. The opposite pattern was observed for unassisted births, which decreased more over time in the CCGs dealing with a single local authority. The triple-difference analyses confirmed that the effects were statistically significant and robust to different model specifications.

For peer review only - http://bmjopen.bmj.com/site/about/guidelines.xhtml

We have demonstrated unintended consequences arising out of a large-scale health system reform. Taken together, our findings suggest that there is an urgent need for clarification as to who holds the budget, and therefore who should be commissioning, cervical screening in the English NHS, and for local agreements to ensure that issues over funding and budgets do not disrupt screening programmes. More broadly, the issues we have identified in this study are of value to policy makers and system leaders in other health systems. The current study suggests that there are particular problems associated with service commissioning where co-ordination is required between multiple commissioners. This suggests that future commissioning reforms should include assessment of the likely impact on co-ordination, and a presumption in favour of commissioning all required services for geographical populations where possible. This may also have implications for mixed health systems, in which multiple payers (including public and private insurers as well as out of pocket payments) are responsible for services. In such systems achieving desirable population coverage for services such as screening may require specific co-ordination efforts.

Potential confounders and study strengths

We took 2009 as our starting point for pre-HSCA cervical screening activity. Two potential confounders to our results were considered. First, the high-profile case of Jade Goody, a 27-year-old reality TV star who was diagnosed with cervical cancer in August 2008 and died in March 2009. The contemporaneous media attention and publicity was linked with a substantial increase in cervical screening rates (around an extra half a million women) during the time between Goody's diagnosis and death. However, previous impacts of high-profile cases of celebrity cancer diagnoses upon population behaviour have tended to be brief and immediate rather than longer-lasting, and, therefore, we are confident that from 2010, rates of cervical screening returned towards underlying trends.[21] Second, the UK's Human Papillomavirus (HPV) vaccination programme was introduced in 2008 in order to reduce the incidence of cervical cancer.[22] The vaccine is offered to all girls aged 12-13 years, and figures for 2008-2014 show high uptake rates of just under 90%. It is likely that this vaccination programme will contribute to a reduction in cervical screening activity in future. However, the first cohort of women in the programme, i.e. those aged 12-13 in 2008, were only 21-22 years old in 2016/17, hence too young to have been invited for routine cervical screening (which begins at age 25) at the time of the study. We can, therefore, be confident that any changes to cervical screening rates cannot yet be attributed directly to the HPV programme, but any future research into cervical screening rates needs to take this into account.

We considered whether the results were sensitive to the group of CCGs in terms of the number of local authorities they worked with. The direction of results was the same, and the strength and

For peer review only - http://bmjopen.bmj.com/site/about/guidelines.xhtml

significance was increased, comparing CCGs working with one or two local authorities with those CCGs working with more than two local authorities. We also considered whether the results were sensitive to the choice of comparison indicator (unassisted births) for maternity services. We tested whether the results would hold for another indicator of maternity services: the rate of deliveries by caesarean section. We observed the same direction and significance of results for this indicator as well.

The average age of mothers at delivery is likely to be younger than the average age of women attending for cervical screening. For our analysis, we require that differential changes in maternity indicators between CCGs with simple and CCGs with complex local authority relationships are a good proxy for other factors influencing cervical screening rates. We have confirmed the empirical validity of this assumption by looking for parallel trends in the period before the HSCA, but we can never be entirely sure of its validity.

The findings presented here come from a longitudinal study of major healthcare system reform conducted by a multidisciplinary research team. The nature of this study facilitated the development of the relatively novel, sequential mixed methods approach in which the claims made in qualitative interviews could be tested in a subsequent quantitative analysis. There is a reinforcing effect in this analytical approach, which provides a strong cumulative indication that in areas of the country where complexity and coordination issues linked to the HSCA were more likely to occur there was an associated reduction in cervical screening rates.

ACKNOWLEDGMENTS

The authors would like to thank the research participants for their involvement, and acknowledge the valuable advice of the Project Advisory Group.

For peer review only - http://bmjopen.bmj.com/site/about/guidelines.xhtml

REFERENCES

[1] Best A, Greenhalgh T, Lewis S, Saul JE, Carroll S, Bitz J. Large-system transformation in health care: a realist review. Milbank Quarterly 2012;90:421–56. doi:10.1111/j.1468-0009.2012.00670.x.

[2] Edwards N. The triumph of hope over experience: Lessons from the history of reorganisation. NHS Confederation; 2010.

[3] Lale AS, Temple JMF. Has NHS reorganisation saved lives? A CuSum study using 65 years of data. J R Soc Med 2016;109:18–26. doi:10.1177/0141076815608853.

[4] Department of Health. The NHS Plan: a plan for investment, a plan for reform. London: The Stationery Office; 2000.

[5] Propper C. Market structure and prices: The responses of hospitals in the UK National Health Service to competition. Journal of Public Economics 1996;61:307–35. doi:10.1016/0047-2727(95)01561-2.

[6] Barbour R. The case for combining qualitative and quantitative approaches in health services research. J Health Serv Res Policy 1999;4:39–43. doi:10.1177/135581969900400110.

[7] Health and Social Care Act. (c.7). London: HMSO: 2012.

[8] Ham C, Baird B, Gregory S, Jabbal J, Alderwick H. The NHS under the coalition government. Part one: NHS reform. London: 2015.

[9] Peckham S, Falconer J, Gillam S, Hann A, Kendall S, Nanchahal K, et al. The organisation and delivery of health improvement in general practice and primary care: a scoping study. National Institute for Health Research; 2015.

[10] Weiss CH. Evaluation: methods for studying programs and policies. New Jersey: Prentice Hall; 1998.

[11] Department of Health. Equity and excellence: liberating the NHS. London: The Stationery Office; 2010.

[12] NHS England, Public Health England. Immunisation and screening national delivery framework & local operating model. 2013.

[13] Peckham S, Gadsby E, Jenkins L, Coleman A, Bramwell D, Perkins N. Views of public health leaders in English local authorities – changing perspectives following the transfer of responsibilities from the National Health Service to local government. Local Government Studies 2017;43:842–63. doi:10.1080/03003930.2017.1322069.

[14] Guest G. Describing mixed methods research: an alternative to typologies. Journal of Mixed Methods Research 2013;7:141–51. doi:10.1177/1558689812461179.

[15] Checkland K, Hammond J, Sutton M, Coleman A, Allen P, Mays N, et al. Understanding the new commissioning system in England: contexts, mechanisms and outcomes. Final report. PR-R6-1113-25001: NIHR Policy Research Programme; 2018.

[16] Braun V, Clarke V. Using thematic analysis in psychology. Qualitative Research in Psychology 2006;3:77–101. doi:10.1191/1478088706qp063oa.

[17] Imbens GW, Wooldridge JM. Recent developments in the econometrics of program evaluation. Journal of Economic Literature 2009;47:5–86. doi:10.1257/jel.47.1.5.

[18] O'Neill S, Kreif N, Grieve R, Sutton M, Sekhon JS. Estimating causal effects: considering three alternatives to difference-in-differences estimation. Health Serv Outcomes Res Methodol 2016;16:1–21. doi:10.1007/s10742-016-0146-8.

[19] Stevens S, Valderas JM, Doran T, Perera R, Kontopantelis E. Analysing indicators of performance, satisfaction, or safety using empirical logit transformation. BMJ 2016;352:i1114. doi:10.1136/bmj.i1114.

[20] Rogers W. sg17: Regression standard errors in clustered samples. Stata Technical Bulletin 1993;3:19–23.

For peer review only - http://bmjopen.bmj.com/site/about/guidelines.xhtml

[21] Lancucki L, Sasieni P, Patnick J, Day T, Vessey M. The impact of Jade Goody's diagnosis and death on the NHS Cervical Screening Programme. J Med Screen 2012;19:89–93. doi:10.1258/jms.2012.012028.
[22] Public Health England. Human Papillomavirus (HPV) Vaccine Coverage in England, 2008/09 to 2013/14. 2015.

FOOTNOTES

Author statement / Contributors: KC designed the study with input from MS, NM, PA, AC. JH, AH, LWG gathered and analysed the qualitative data. TM and MS designed the quantitative evaluation and conducted this analysis. JH drafted the manuscript to which all authors made substantial contributions. All authors approved the final version and agree to be accountable for all aspects of the analysis.

Funding: The report is based on independent research commissioned and funded by the NIHR Policy Research Programme ('Understanding the new commissioning system in England: contexts, mechanisms and outcomes', PR-R6-1113-25001). The views expressed in the publication are those of the author(s) and not necessarily those of the NHS, the NIHR, the Department of Health, arm's-length bodies or other government departments.

Competing interests: None declared.

Patient consent: Not required.

Ethics approval: Ethical approval was granted by one of The University of Manchester Research Ethics Committees (application 15085) in March 2015. Participants were provided written information about the study, provided written consent, or gave consent verbally at the beginning of telephone interviews.

Data sharing statement: No additional data are available.

For peer review only

For peer review only - http://bmjopen.bmj.com/site/about/guidelines.xhtml

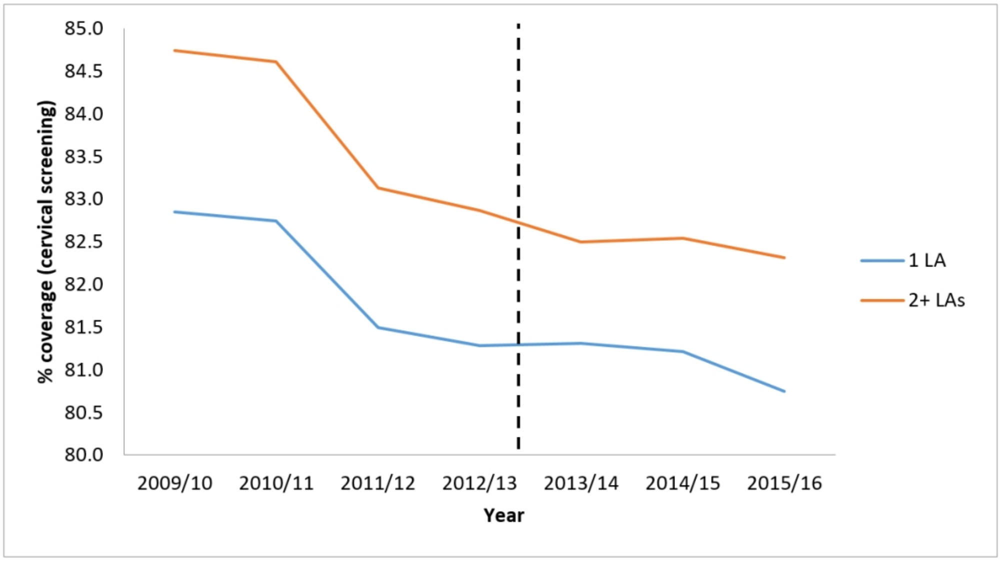

Figure 1: Uptake (%) of cervical screening pre and post HSCA

112x62mm (300 x 300 DPI)

Technical Appendix

Algebraic expressions for the estimated models

Triple-difference model

The triple-difference model can be written algebraically as:

$$\boldsymbol{y_{cjt} = \alpha + \phi L_j + \theta \pi_c + \rho L_j \pi_c + \sum_{t=2}^{T} \delta_t + \sum_{t=2}^{T} \psi_t \pi_c + \sum_{t=2}^{T} \gamma_t L_j + \beta L_j \pi_c D_t^* + \varepsilon_{cjt}}$$

in which y_{cjt} is the value of indicator c in practice j and year t, L_j is a binary indicator for practices located in exposed areas, π_c is a dummy variable for the intervention indicator, $t_2 \ldots t_T$ are dummy variables for years, and $\boldsymbol{D_t^*}$ is a dummy variable taking a value of one in post-implementation period. The coefficient of interest is β, the coefficient on the three-way interaction between the exposed area, the intervention indicator and the post-implementation period.

Lagged Dependent Variable model

The Lagged Dependent Variable model can be written algebraically as:

$$\boldsymbol{y_{cjt} = \alpha + \phi L_j + x \pi_c + \rho L_j \pi_c + \sum_{t=t^*}^{T} \delta_t + \sum_{t=t^*}^{T} \psi_t \pi_c + \sum_{k=1}^{t^*-1} \mu_k y_{cjk} + \sum_{k=1}^{t^*-1} \vartheta_k \pi_c y_{cjk} + \varepsilon_{cjt}}$$

in which y_{cjt} is the value of indicator c in practice j and year t, L_j is a binary indicator for practices located in exposed areas, π_c is a binary indicator for the intervention indicator, and y_{cjk} are lagged values of the dependent variable in the pre-period ending at $t=t^*-1$. The model is estimated on data from the post period only and the ρ coefficient on the $L_j\pi_c$ term is the coefficient of interest.

For peer review only
